# Steric interference from intrinsically disordered regions controls dynamin-related protein 1 self-assembly during mitochondrial fission

**DOI:** 10.1038/s41598-018-29001-9

**Published:** 2018-07-18

**Authors:** Bin Lu, Bridget Kennedy, Ryan W. Clinton, Emily Jue Wang, Daniel McHugh, Natalia Stepanyants, Patrick J. Macdonald, Jason A. Mears, Xin Qi, Rajesh Ramachandran

**Affiliations:** 1 Department of Physiology & Biophysics, Case Western Reserve University School of Medicine, Cleveland, OH 44106 USA; 2 Department of Pharmacology, Case Western Reserve University School of Medicine, Cleveland, OH 44106 USA; 3 Center for Mitochondrial Diseases, Case Western Reserve University School of Medicine, Cleveland, OH 44106 USA; 4 Cleveland Center for Membrane and Structural Biology, Case Western Reserve University School of Medicine, Cleveland, OH 44106 USA

## Abstract

The self-assembling, mechanoenzymatic dynamin superfamily GTPase, dynamin-related protein 1 (Drp1), catalyzes mitochondrial and peroxisomal fission. Distinct intrinsically disordered regions (IDRs) in Drp1 substitute for the canonical pleckstrin homology (PH) domain and proline-rich domain (PRD) of prototypical dynamin, which cooperatively regulate endocytic vesicle scission. Whether the Drp1 IDRs function analogously to the corresponding dynamin domains however remains unknown. We show that an IDR unique to the Drp1 GTPase (G) domain, the ‘extended 80-loop’, albeit dissimilar in location, structure, and mechanism, functions akin to the dynamin PRD by enabling stable Drp1 mitochondrial recruitment and by suppressing Drp1 cooperative GTPase activity in the absence of specific partner-protein interactions. Correspondingly, we find that another IDR, the Drp1 variable domain (VD), in conjunction with the conserved stalk L1N loop, functions akin to the dynamin PH domain; first, in an ‘auto-inhibitory’ capacity that restricts Drp1 activity through a long-range steric inhibition of helical inter-rung G-domain dimerization, and second, as a ‘fulcrum’ for Drp1 self-assembly in the proper helical register. We show that the Drp1 VD is necessary and sufficient for specific Drp1-phospholipid interactions. We further demonstrate that the membrane-dependent VD conformational rearrangement essential for the alleviation of Drp1 auto-inhibition is contingent upon the basal GTP hydrolysis-dependent generation of Drp1 dimers from oligomers in solution. IDRs thus conformationally couple the enzymatic and membrane activities of Drp1 toward membrane fission.

## Introduction

Dynamin superfamily proteins (DSPs) comprise a diverse collection of large, modular GTPases related by structure as well as by function^1–3^. With a few noted exceptions, the vast majority of this superfamily functions to catalyze various membrane remodeling events in the cell ranging from the scission of small transport vesicles from donor membrane compartments to the fission and fusion of whole organelles such as the mitochondria, peroxisomes and chloroplasts. DSPs that mediate membrane fission do so via the formation of helical, oligomeric scaffolds that dynamically enwrap and mechanoenzymatically constrict target membranes upon GTP hydrolysis^3–8^.

Broadly grouped into ‘classical dynamins’ and ‘dynamin-related proteins’ (DRPs) based on distinctive variations in structure, it was until recently believed that the classical dynamins primarily function in the scission of small vesicles (≤100-nm diameter) from parent membranes, whereas the DRPs predominantly catalyze the fission (division) of significantly large organelles (>500-nm in diameter)^1,2^. Emergent data however appear to dispel this notion. Although the precise molecular mechanisms are still unknown, a classical dynamin (dynamin 2) was recently reported to be critically involved in mitochondrial fission^9^, whereas a DRP (dynamin-related protein 1 or Drp1) was previously implicated in synaptic vesicle scission^10^. How classical dynamins and DRPs, which are indispensable for numerous membrane fission events in the cell, remain functionally distinct during such disparate membrane remodeling processes is unclear.

**Figure 1:**
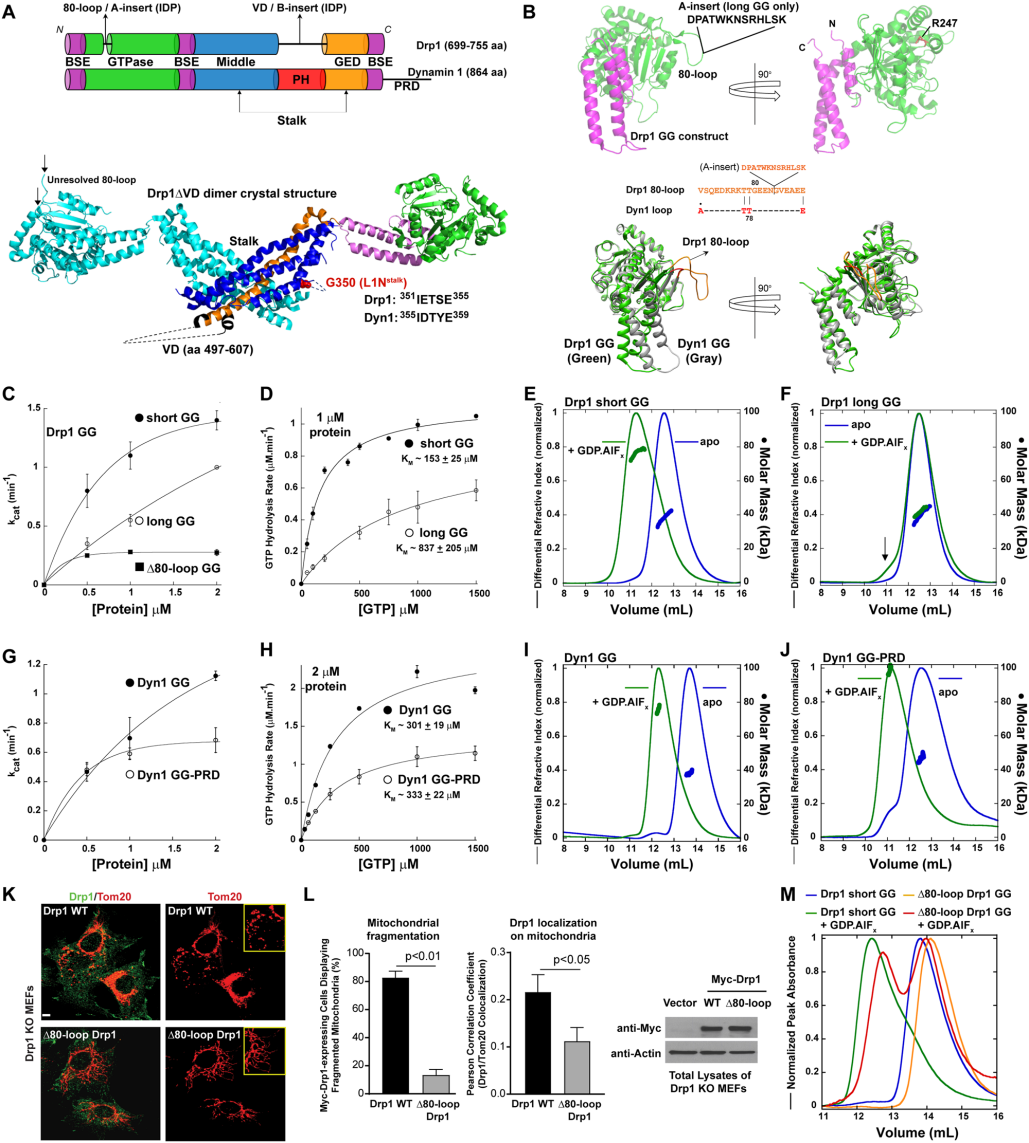
The Drp1 80-loop functions akin to the Dyn1 PRD. (**A**) *Top*, color-coded domain organization of Drp1 relative to Dyn1. The relative positions of the Drp1-specific 80-loop and the VD are indicated. BSE; bundle signaling element, PH; pleckstrin homology domain, and GED; GTPase effector domain. *Bottom*, crystal structure of the Drp1ΔVD dimer (PDB ID: 4BEJ), with one monomer color-coded in correspondence to the primary structure (top panel), showing the relative positions of the VD and the loop 1 N (L1N) of the stalk. High conservation of the Drp1 and Dyn1 L1N sequence is denoted. (**B**) *Top*, color-coded 3D structure of Drp1 short GG (PDB ID: 4H1U) shown in two different orientations to highlight the relative positions of the 80-loop and residue R247. The 13 additional aa residues of the 80-loop that constitutes the ‘A-insert’ of Drp1-long, not present in the structure, is denoted. *Bottom*, An overlay of the Drp1 GG (green) and Dyn1 GG (gray; PDB ID: 2X2E) crystal structures in two different orientations depicting the conspicuous protuberance of the Drp1-specific 80-loop (orange) relative to the corresponding segment in Dyn1 (red). Alignment of the Drp1 80-loop sequence with the corresponding segment of Dyn1 showing conservation of the two consecutive T residues essential for the β-turn that connects the two β-strands. The position of the A-insert sequence that extends the 80-loop in Drp1-long is denoted. (**C**) Basal GTPase activity of Drp1 short GG relative to Drp1 long GG and Δ80-loop GG as a function of protein concentration. *k*_*cat*_ is the turnover number in min^−1^. (**D**) Basal GTPase activities of Drp1 short GG and Drp1 long GG as a function of GTP concentration. The Michaelis constant (*K*_*M*_) was determined by fitting the kinetic data to the Michaelis-Menten equation. Data shown are an average of three independent experiments ± SD. (**E**,**F**) SEC-MALS profiles of Drp1-short GG (D) and Drp1-long GG (**E**) (~40 kDa as monomers) each loaded at 25 μM onto a Superdex 75 10/300 GL column in the absence and presence of the transition-state analog, GDP.AlF_x_. Arrow in (**F**) points to a relatively small dimer population. (**G)** Basal GTPase activities of Dyn1 GG and Dyn1 GG-PRD as a function of protein concentration. (**H**) Michaelis-Menten kinetics of Dyn1 GG and Dyn1 GG-PRD. (**I**,**J**) SEC-MALS profiles of Dyn1 GG (**I**) and Dyn1 GG-PRD (**J**) (~40 kDa as monomers) each loaded at 15 μM onto a Superdex 75 10/300 GL column in the absence and presence of the transition-state analog, GDP.AlF_x_. (**K**) Confocal fluorescence images of mitochondrial morphology in Drp1 KO MEFs expressing either Myc-tagged Drp1 WT (top panels) or Δ80-loop Drp1 (bottom panels) stained for both Drp1 (green; left panels) and mitochondria (red). *Insets* show fragmented mitochondria in the case of Drp1 WT, or hyperfused mitochondrial networks in the case of Δ80-loop Drp1. (**L**) (*left panel*) Quantification of mitochondrial fragmentation in Drp1 KO MEFs cells expressing either Myc-tagged Drp1 WT or Δ80-loop Drp1. (*middle panel*) Co-localization of Drp1 WT and Δ80-loop Drp1, respectively, with mitochondria were determined from confocal fluorescence imaging using the Pearson correlation coefficient. (*right panel*) Representative western blots showing expression levels of Myc-tagged Drp1 WT and Δ80-loop Drp1 in transfected Drp1 KO MEFs. Empty vector-expressing cells served as negative control, and actin was used as loading control for total protein. (**M**) SEC profiles of Drp1 short GG in comparison to Drp1 Δ80-loop Drp1 each loaded at 6 μM onto a Superdex 75 10/300 GL column in the absence and presence of the transition-state analog, GDP.AlF_x_.

The archetypal DRP, Drp1, primarily resides in the cytosol akin to the classical dynamins, but is transiently recruited to the outer membrane of mitochondria and to peroxisomes at the onset of membrane fission^11–13^. Ancient in evolutionary origin in relation to the classical dynamins^14^, Drp1 is distinguished by the presence of various unique intrinsically disordered polypeptide regions (IDRs) that substitute for the canonical pleckstrin homology (PH) domain and the proline-rich domain (PRD) of dynamin (Fig. 1A). The dynamin PH domain and PRD participate in protein-membrane and protein-protein interactions, respectively^4,7^. Whether the Drp1 IDRs function analogously remains unknown.

The first IDR region in Drp1 is the Drp1-specific ‘80-loop’ (residues 69–89 in the ubiquitous Drp1 isoform^8^), which is present within the N-terminal GTPase (G) domain sequence and extends from the globular G-domain fold as a conspicuous protuberance (Fig. 1A,B)^8,15^. As expected for an IDR with inherent flexibility and dynamics, the 80-loop remains unresolved in the available high-resolution crystal and cryo-EM structures of Drp1 (Fig. 1A,B)^16–19^. In select Drp1 splice variants, this 80-loop is further extended by an additional 13–19 amino acid residues called the ‘A-insert’ to generate an ‘extended 80-loop’^12,15,20^ (Fig. 1A,B). Recent data suggest that the maximally extended Drp1 80-loop also participates in the targeting of Drp1 to late endosomes, lysosomes, and the plasma membrane, indicating that this IDR may function as a recognition motif for specific binding partners located at the various membranes^20^.

The second Drp1 IDR region is significantly longer, ranging between ~111–148 aa residues in length also as a result of alternative splicing, and occurs in place of the dynamin PH domain, originating at the membrane-proximal base of the Drp1 molecule (Fig. 1A)^8,15,16^. This region is termed the ‘variable domain’ or ‘VD’, which also contains an alternatively spliced 37-aa polypeptide insert, otherwise called the ‘B-insert’, in select splice variants (Fig. 1A). Likewise, there are no high-resolution structures available yet for this region, and is neither present in the crystal structure of nearly full-length Drp1 (Drp1ΔVD)^16^ nor sufficiently resolved in the recent cryo-EM structures of full-length Drp1^18,19^.

In addition to these unique IDRs, Drp1 also contains various conserved loops that connect the bundled helices of the central ‘stalk’ and create interfaces for higher-order helical self-assembly (Fig. 1A)^21^. One such loop, L1N (Fig. 1A), lies in close proximity to the PH domain-stalk interface in dynamin^22–24^, and is predicted to conformationally couple PH domain-membrane interactions to higher-order dynamin self-assembly^22,23^. Although L1N is highly conserved between dynamin and Drp1 (Fig. 1A), whether a similar mechanism exists in Drp1, which contains the disordered VD in place of the PH domain, and interfaces with membrane-anchored protein adaptors^19^, is unknown.

The PH domain is both necessary and sufficient for specific dynamin interactions with phosphatidylinositol-4, -5-bisphosphate (PIP_2_), essential for endocytic vesicle scission at the plasma membrane^25–28^. Whether the corresponding VD in Drp1 is necessary or sufficient for specific interactions with the mitochondrial target phospholipid, cardiolipin (CL) however remains unclear^29–32^. Similarly, the PRD regulates dynamin GTPase activity and mediates interactions with various protein partners that recruit dynamin to the plasma membrane and cooperatively regulate endocytic vesicle scission^7,33,34^. Whether the Drp1-specific 80-loop functions analogously to the dynamin PRD to regulate essential Drp1 interactions during mitochondrial fission is yet to be determined^11,15,32,35,36^.

In this elaborate study, we first compare and contrast the Drp1 and Dyn1 GTPase domains in isolation, and in the absence and presence of the 80-loop/A-insert and the PRD, respectively, to show that the Drp1-specific ‘extended 80-loop’ functions equivalently to the dynamin PRD, in an auto-inhibitory capacity, by suppressing G-domain dimerization-dependent cooperative GTPase activity. Secondly, we reveal for the first time, the molecular basis of the previously identified VD auto-inhibition of Drp1 activity^31^. We identify the presence of an intramolecular interaction site for the Drp1 VD and demonstrate that VD interactions with this site sterically interfere with helical inter-rung G-domain dimerization essential for cooperative GTPase activity. Using the isolated Drp1 VD, we show that the VD is necessary and sufficient for specific CL interactions. The VD, upon stable membrane interactions, also appears to function as a ‘fulcrum’ for Drp1 self-assembly in a defined helical register. Lastly, we establish that the VD conformational rearrangement essential for the alleviation of its auto-inhibitory activity is contingent upon the basal GTP hydrolysis-driven generation of Drp1 dimers from oligomers in solution. Collectively, these results establish that the molecular mechanisms that govern dynamin and Drp1 activities in intracellular membrane remodeling events are comparable and evolutionarily conserved.

## Results

### The Drp1 80-loop/A-insert functions akin to the Dyn1 PRD

Cooperative GTP hydrolysis in both dynamin and Drp1 depends on nucleotide-dependent intermolecular G-domain dimerization, which increases with increasing protein concentration, and is facilitated conceivably by stalk-mediated higher-order self-assembly on target membranes^35,37,38^. Using the previously described minimal and monomeric GTPase-GED (GG) construct of Drp1 (Drp1 GG)^17^ (Fig. 1B), we first explored whether the basal GTPase activity of Drp1 stems from enzymatic cooperativity even in the absence of stalk-mediated higher-order self-assembly. Consistent with this notion, the basal GTPase activity (*k*_*cat*_) of Drp1 GG (short splice variant^15^; hereafter referred to as short GG), when examined at physiologically relevant low micromolar concentrations (≤2 μM) in solution, increased with increasing protein concentration (Fig. 1C). These data indicated that Drp1 basal GTPase activity is enzymatically cooperative and depends on concentration-dependent G-domain self-interactions in solution.

To ascertain this unexpected finding, we characterized a longer variant of the Drp1 GTPase domain (long GG) containing a 13-aa-residue A-insert within the 80-loop (Fig. 1C). We previously showed that the basal GTPase activity of full-length Drp1-long containing the A-insert was significantly lower than that of Drp1-short^15^. We therefore examined whether the A-insert directly suppressed Drp1 cooperative GTPase activity even in the absence of stalk-mediated higher-order self-assembly. Remarkably, the basal GTPase activity of long GG was still significantly lower than that of short GG, and again increased linearly with increasing protein concentration. These data indicated that G-domain dimerization in Drp1-long was less cooperative compared to Drp1 short, and that the A-insert directly auto-inhibited Drp1-long cooperative GTPase activity. Moreover, the K_M_ of cooperative GTP hydrolysis in long GG was significantly higher than that of short GG indicating a patent auto-inhibition imposed by the A-insert (Fig. 1D). Not surprisingly, the K_M_ of GTP hydrolysis determined for the short and long GG variants closely matched that of the corresponding full-length Drp1 variants (290 ± 40 μM and ~1000 ± 300 μM for Drp1-short 80-loop and Drp1-long-80-loop, respectively)^15^. We conclude from these data that Drp1 G-domain dimerization and cooperative GTP hydrolysis, while conceivably promoted by stalk–stalk interactions, do not necessarily rely on stalk-mediated higher-order self-assembly.

We previously demonstrated that both full-length Drp1-short and Drp1-long bind GTP with indistinguishable affinities^15^. Therefore, to more precisely define the molecular basis of the A-insert-imposed auto-inhibition on Drp1-long GTPase activity, we assessed whether the A-insert somehow dynamically impaired the transition state-dependent dimerization of the Drp1 GTPase domain^17^. To this end, we used size-exclusion chromatography (SEC)-coupled multi-angle light scattering (MALS) to assess the oligomeric states of the short and long GG variants in the absence and presence of the transition-state mimic, GDP.AlF_x_. In agreement with previous reports^17^, short GG, which was exclusively monomeric in the absence of nucleotide, formed stable dimers in the presence of GDP.AlF_x_ (Fig. 1E). By contrast, long GG remained largely monomeric in solution, with only a semblance of dimers (Fig. 1F). From these data, we conclude that the A-insert functions to suppress Drp1 cooperative GTPase activity by dynamically auto-inhibiting transition state-dependent G-domain dimerization.

Dyn1, unlike Drp1, does not contain a disordered 80-loop/A-insert-like region within its G domain (Fig. 1A,B). However, Dyn1 contains an IDR in the form of the PRD (residues 750–864), which extends from the C-terminal helix of the BSE, and is located apparently in close proximity to the GTPase domain in the Dyn1 3D structure^39^. No high-resolution structures of the PRD are yet currently available. Regardless, the Dyn1 PRD was previously determined to be a positive regulator of Dyn1 self-assembly^40,41^, and more recently also demonstrated to be a negatively regulator of cooperative GTPase activity, when swapped into the more enzymatically cooperative Dyn2 and Dyn3 isoforms^34^. Therefore, we addressed the possibility that the Dyn1 PRD plays a role akin to the Drp1 80-loop/A-insert by directly suppressing Dyn1 cooperative GTPase activity, even in the absence of stalk-mediated self-assembly, as observed for Drp1.

To this end, we newly constructed minimal Dyn1 GG and Dyn1 GG-PRD constructs comparable to that of Drp1 (see Methods), and examined whether the presence of the PRD auto-inhibited Dyn1 GG GTPase activity akin to the effect of the A-insert on Drp1-long GG. Again, unexpectedly, the catalytic activity (*k*_*cat*_) of Dyn1 GG increased with increasing protein concentration (under 2 μM) suggesting that the basal GTPase activities of Dyn1 and Drp1 are both enzymatically cooperative and are likely dependent on concentration-dependent G-domain dimerization (Fig. 1G)^42^. Remarkably, the presence of the PRD robustly suppressed cooperative GTPase activity in Dyn1 GG-PRD suggesting that the Dyn1 PRD functions akin to the Drp1 A-insert as a negative regulator of cooperative GTP hydrolysis (Fig. 1G). However, unlike for the Drp1 short- and long-GG constructs, no significant difference in the *K*_*M*_ of GTP hydrolysis was observed between Dyn1 GG and Dyn1 GG-PRD (Fig. 1H). These data indicated that the mechanisms of auto-inhibition imposed by the Drp1 A-insert and the Dyn1 PRD on the respective G domains are however disparate. Consistently, SEC-MALS analyses of Dyn1 GG and Dyn1 GG-PRD also revealed no difference in their respective dimerization properties in the presence of GDP.AlF_x_ (Fig. 1I,J).

Remarkably comparable effects were also observed for the respective Drp1 and Dyn1 full-length variants (Supplementary Fig. S1). As previously shown^15^, the basal GTPase activity of Drp1-short-80-loop exhibited greater cooperativity relative to Drp1-long-80-loop, when tested as a function of protein concentration (Supplementary Fig. S1A). This effect was further exacerbated under low ionic-strength conditions that promote Drp1 higher-order self-assembly even in the absence of a target membrane template (Supplementary Fig. S1B). Also consistent with our previous findings^15^, the lipid-stimulated cooperative GTPase activity of Drp1-short-80-loop on CL-containing liposomes was substantially greater than that of Drp1-long-80-loop (Supplementary Fig. S1C). Notably, the steep dependence of CL-stimulated GTPase activity (*k*_*cat*_) on Drp1 concentration^35^ (Supplementary Fig. S1C) was comparable to that of Dyn1 on PIP_2_-containing liposomes reported previously^43^. These data indicated that Drp1 and Dyn1 both exhibit V-type allostery^43,44^, wherein the equilibrium is shifted in favor of enzymatically cooperative higher-order polymers as a function of protein concentration.

Consistent differences nevertheless emerged upon comparison of full-length Dyn1 with Dyn1ΔPRD. No substantial difference in the basal GTPase activities of Dyn1 and Dyn1ΔPRD were observed, although both increased likewise as a function of protein concentration (Supplementary Fig. S1D). However, when examined at low ionic-strength, the assembly-stimulated GTPase activity of Dyn1 was substantially greater than that of Dyn1ΔPRD (Supplementary Fig. S1E), likely owing to the presence of the higher-order self-assembly-promoting PRD^40,41^. Remarkably, however, the assembly-stimulated GTPase activity of full-length Dyn1 decreased with increasing protein concentration indicating negative cooperativity, whereas that of the Dyn1ΔPRD increased with increasing protein concentration conversely indicating positive cooperativity (Supplementary Fig. S1E). From these data, we conclude that the Dyn1 PRD, while positively regulating Dyn1 self-assembly, functions equivalently to the Drp1 A-insert as a negative regulator of cooperative GTPase activity upon higher-order Dyn1 self-assembly.

The PRD plays an essential role in dynamin recruitment to endocytic pits for fission^45–47^. Given their apparent functional equivalence, we next explored whether the 80-loop/A-insert region might likewise be important for Drp1 recruitment to the mitochondrial surface. Therefore, we generated a Drp1 deletion mutant lacking the 80-loop (Δ80-loop Drp1). A pair of Thr residues completes the β-turn in the Drp1 80-loop, and is conserved between Drp1 and Dyn1 (Fig. 1B). Conserving these two Thr residues essential for connecting the two β-strands, we deleted the remainder of the 80-loop sequence to generate Δ80-loop Drp1, now structurally comparable to Dyn1 in the GTPase domain. Remarkably, when expressed in Drp1-*null* mouse embryonic fibroblasts (Drp1 KO MEFs) as earlier^35,48^, Δ80-loop Drp1, unlike Drp1 WT, was unable to restore mitochondrial fission (Fig. 1K,L (left panel)). More surprisingly, when compared to Drp1 WT, mitochondrial recruitment of Δ80-loop Drp1 was significantly impaired (Fig. 1L (middle panel)), despite being expressed to comparable levels (Fig. 1L (right panel), Supplementary Fig. S2). These data indicated a role for the 80-loop in Drp1 mitochondrial recruitment akin to that of the PRD essential for Dyn1 recruitment to the plasma membrane via PRD-partner protein interactions^49^. Consistent with our findings, recent studies have identified a role for the extended 80-loop in Drp1 recruitment also to extra-mitochondrial membranes, such as the late endosomes, lysosomes and the plasma membrane^20^.

We tested whether one such binding partner for the 80-loop might be the partnering GTPase domain of an enzymatically cooperative G-domain dimer, as alluded to by recent X-ray crystallographic and cryo-EM studies^17,18^. Indeed, SEC analyses of Δ80-loop Drp1 GG in the absence and presence of GDP.AlF_x_ revealed a marked impairment in transition state-dependent G-domain dimerization and a substantially lower cooperative GTPase activity relative to Drp1 short GG WT (Fig. 1C,M). Notably, the degree of impairment in G-domain dimerization in Δ80-loop Drp1 GG correlated, at least qualitatively, with the degree of impairment of Δ80-loop Drp1 recruitment to the mitochondrial surface (Fig. 1L). These data suggested that a stabilization of the preferentially recruited cytosolic Drp1 dimers on the mitochondrial surface via the 80-loop-promoted *in trans* dimerization interactions of the GTPase domain facilitates Drp1 polymerization for fission^18,35^. Consistent with this interpretation, unlike Drp1 WT, full-length Δ80-loop Drp1 was unable to constitute helical oligomers either in the presence of the non-hydrolyzable GTP analog, GMP-PCP, in solution, or upon incubation with membrane templates containing the target phospholipid, CL. These specific impairments also coincided with a markedly reduced GTPase activity relative to WT under either condition (Fig. 1C, Supplementary Fig. S3).

Combined together, these data reveal that although structurally and mechanistically disparate, the Drp1 80-loop/A-insert region functions equivalently to the Dyn1 PRD, both as a positive regulator of self-interactions, as demonstrated for Dyn1 previously^40,41^, and as a negative regulator of cooperative GTPase activity as established here.

### Helical Drp1 self-assembly is not essential for cooperative GTPase activity

As established previously, full-length Drp1 constitutes helical polymers both in solution and on membranes^35^. The helical self-assembly of Drp1 is posited to bring the G domains of adjacent helical rungs in close proximity to effect nucleotide-dependent, inter-rung G-domain dimerization and cooperative GTPase activity^37,50^. However, given that Drp1 nucleotide-dependent G-domain dimerization and cooperative GTP hydrolysis can occur independently of the rest of the molecule (Fig. 1), we surmised that helical self-assembly, or organized lattice formation, itself might not be critical for cooperative GTPase activity, even in the case of full-length Drp1.

**Figure 2:**
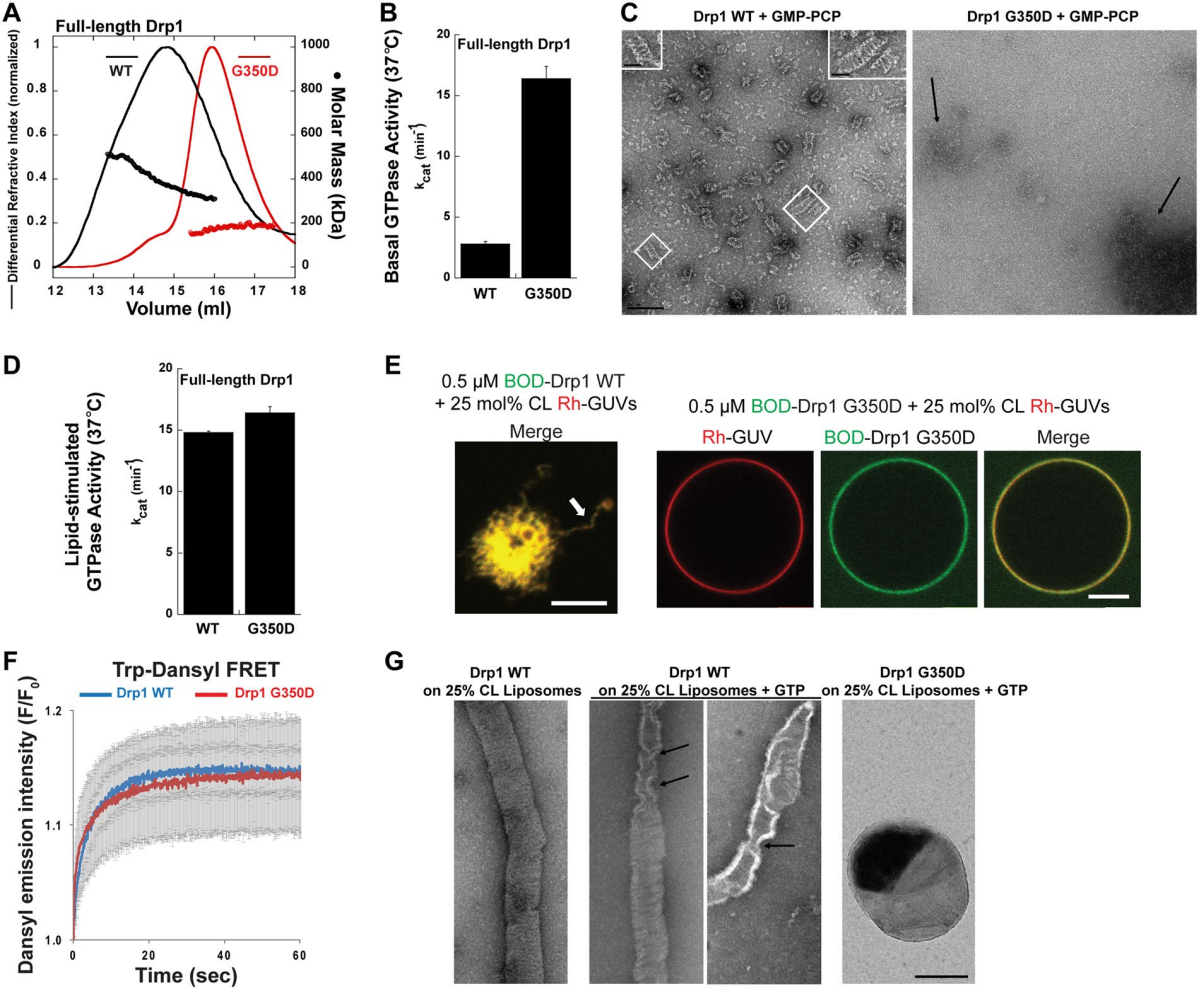
Drp1 helical self-assembly is not essential for cooperative GTPase activity. (**A**) SEC-MALS profiles of full-length Drp1 WT and Drp1 G350D each loaded at 10 μM onto a Superose 6 10/300 GL column. (**B**) The basal GTPase activity of Drp1 G350D relative to Drp1 WT was measured in the absence of target membranes at 37 °C in solution. (**C**) Representative EM images of Drp1 WT and Drp1 G350D polymers formed in the presence of GMP-PCP in solution. Scale bar, 200 nm. *Inset*, magnified images of helical Drp1 WT polymers. *Inset* scale bar, 50 nm. (**D**) Lipid-stimulated GTPase activity of Drp1 G350D relative to Drp1 WT was measured at 37 °C upon incubation with 25 mol% CL-containing liposomes. (**E**) Representative confocal images of RhPE-labeled, 25 mol% CL-containing GUVs (red) upon incubation with BODIPY-FL-labeled (BOD) Drp1 WT or Drp1 G350D (green). Both merged and individual color panels are shown for Drp1 G350D. Only the merged color panel is shown for Drp1 WT (left). Extensive membrane tubulation (arrow) is observed for Drp1 WT in contrast to Drp1 G350D. Scale bar, 5 μm. (**F**) Trp-dansyl FRET-sensitized increase in dansyl emission intensity upon Drp1 Trp excitation is plotted as F/F_0_. F_0_ is the background dansyl emission intensity prior to Drp1 addition, and F is dansyl emission intensity at time ‘t’ after Drp1 addition. Representative traces with error bars are shown. (**G**) Representative EM images of Drp1 WT-decorated membrane tubes drawn from 25 mol% CL-containing liposomes by Drp1 helical self-assembly, prior to (*left panel*) and after addition of GTP for 2 min (*middle two panels*), shown in comparison to Drp1 G350D-bound, non-deformed liposomes in the presence of GTP for 2 min (*right panel*). Arrows point to local membrane tube constriction effected by Drp1 WT upon GTP hydrolysis. Scale bar, 200 nm.

We tested this hypothesis by further characterizing a previously described Drp1 stalk mutant, Drp1 G350D (Fig. 1A) that similar to Dyn1 R399A was shown to be defective in higher-order self-assembly^51,52^. SEC-MALS analysis revealed that nucleotide-free Drp1 G350D, similar to Dyn1 R399A, is predominantly dimeric in solution (Fig. 2A). Drp1 WT, by contrast, displayed dynamic dimer-tetramer-oligomer equilibria as previously shown^35^. Surprisingly however, under physiological ionic strength and temperature (37 °C), Drp1 G350D exhibited a substantially higher basal GTPase activity compared to Drp1 WT (Fig. 2B). Negative-stain EM analysis in the presence of the non-hydrolyzable GTP analog, GMP-PCP, revealed that Drp1 G350D, in contrast to Drp1 WT, does not constitute ordered helical polymers in solution (Fig. 2C). Furthermore, CL-containing liposomes over which Drp1 WT spontaneously assembles into helical polymers and exhibits stimulated GTPase activity, did not further stimulate Drp1 G350D basal GTPase activity (Fig. 2D). Notably, the relatively high basal GTPase activity of Drp1 G350D was comparable to the liposome-stimulated GTPase activity of Drp1 WT. These data suggest that a transient, GTP-dependent, non-helical aggregation of Drp1 G350D dimers either in solution, or on membranes, is sufficient to elicit maximal cooperative GTPase activity. Confocal fluorescence imaging of fluorescently labeled Drp1 G350D on CL-containing Giant Unilamellar Vesicles (GUVs) revealed that Drp1 G350D, in contrast to Drp1 WT, does not tubulate membranes via helical self-assembly (Fig. 2E), despite equal membrane binding as measured by Förster resonance energy transfer (FRET) between Drp1 Trp and dansyl-labeled phospholipids distributed randomly in the membrane as previously shown^35^ (Fig. 2F). EM analyses further revealed that Drp1 G350D, in contrast to Drp1 WT, also does not constrict membranes upon GTP hydrolysis (Fig. 2G). We conclude that Drp1 cooperative GTP hydrolysis may occur independently of Drp1 helical self-assembly. These data, together with previous results^36^, caution against the use of GTPase activity assays as a readout for proper Drp1 helical self-assembly.

### The Drp1 VD acts as a fulcrum to direct helical self-assembly

The PH domain of dynamin, in the absence of target PIP_2_ interactions, functions as a negative regulator of stalk-mediated helical self-assembly via the steric, auto-inhibitory ‘masking’ of a critical stalk ‘interface 3’ located at the membrane-proximal base of the molecule^22–24^. Whereas mutations in interface 3, e.g., Dyn1 R399A, specifically disrupt helical self-assembly^52^, mutations at the PH domain-stalk interface alleviate the PH domain-mediated auto-inhibition resulting in premature dynamin higher-order self-assembly and cooperative GTPase activity in solution^53,54^.

Deletion of the PH domain in its entirety, however, has produced confounding results^24,55–57^. Variants of the Dyn1ΔPH construct have been shown to either promote the premature self-assembly of dynamin into higher-order oligomers, or contradictorily, limit dynamin self-assembly in solution. Likewise, deletion of the corresponding VD in Drp1 (Drp1ΔVD), has been controvertibly shown to either promote or limit premature Drp1 self-assembly in solution^29,31,36^.

**Figure 3:**
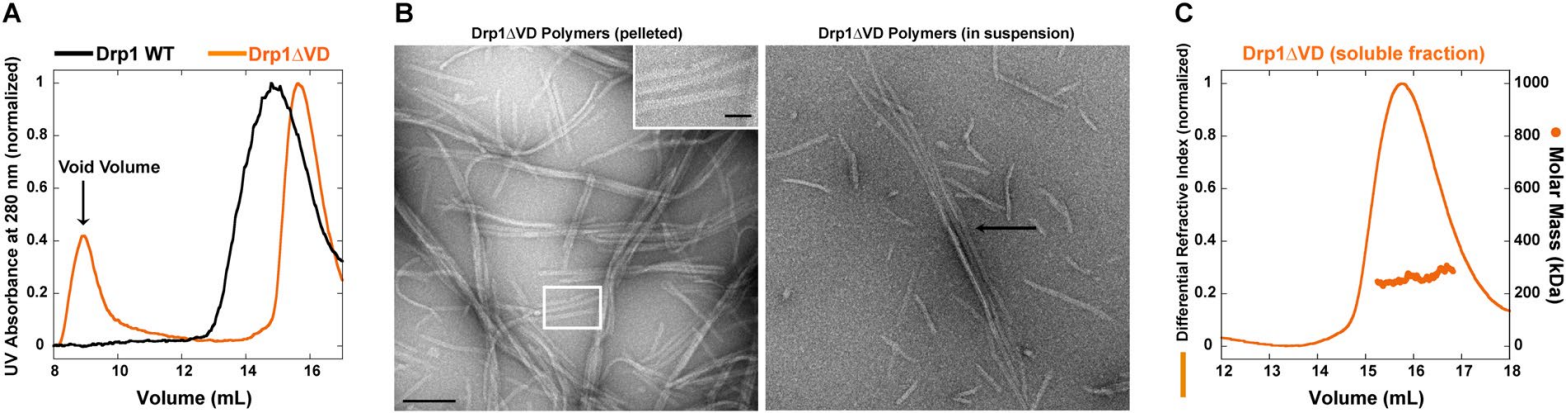
Drp1 VD directs helical self-assembly. (**A**) SEC elution profiles of purified Drp1 WT and Drp1ΔVD each loaded at 6 μM onto a Superose 6 10/300 GL column. (**B**) Representative EM images of Drp1ΔVD polymers upon spin-sedimentation at 20,800×g (left) and in suspension (right). Scale bar, 200 nm. *Inset*, a magnified image of non-helical Drp1ΔVD polymers. Scale bar, 50 nm. (**C**) SEC-MALS profiles of the included fraction of Drp1ΔVD (~70 kDa monomer) loaded at 6 μM onto a Superose 6 10/300 GL column. SEC-MALS revealed that Drp1ΔVD formed tetramers (~70 × 4 = ~280 kDa) in solution.

To dissect the role of the Drp1 VD, and by extension, the dynamin PH domain, we reassessed the biophysical properties of Drp1ΔVD. Although Drp1ΔVD purifies as a soluble protein under high ionic strength conditions as previously shown^36,48^, we noted that a significant fraction of Drp1ΔVD, over time and at physiological salt concentration (150 mM KCl), formed visible aggregates in solution. SEC analysis revealed that the aggregates, which eluted in the void volume, were substantially larger than the oligomers constituted by Drp1 WT, as well as the remainder soluble fraction of Drp1ΔVD (Fig. 3A). Negative-stain EM analysis revealed that this aggregate fraction represented curvilinear filaments of Drp1ΔVD, which approximated ~18 nm in thickness (Fig. 3B, left) and were reminiscent of filaments observed for full-length Drp1 in the presence of the partner protein, MiD49^58^. Furthermore, these filaments were self-organized into lateral arrays and bundles (Fig. 3B), which again were highly reminiscent of Drp1ΔVD bundles previously observed *in vivo*^29^. Together, these data reaffirm that the VD, akin to the dynamin PH domain, functions as a negative regulator of higher-order Drp1 self-assembly. These data also suggests that a VD conformational rearrangement in full-length Drp1, mimicked here by Drp1ΔVD, permits both radial and longitudinal self-interactions in the Drp1 helical scaffold. This was further evidenced by SEC-MALS analysis of the remainder soluble Drp1ΔVD fraction, which was predominantly composed of tetramers constituted from minimal dimers in solution (Fig. 3C)^36^. Of importance, the absence of helical polymerization in Drp1ΔVD suggests that the VD, akin to the dynamin PH domain, functions as a steric constraint, in the form of a fulcrum or a pivot, to direct Drp1 self-assembly in the proper helical register. The previously demonstrated inability of Drp1ΔVD to constrict CL-containing liposomes upon membrane adsorption^31,48^ is therefore primarily a defect of helical self-assembly.

### The Drp1 VD is monomeric and does not participate directly in Drp1 self-assembly

**Figure 4:**
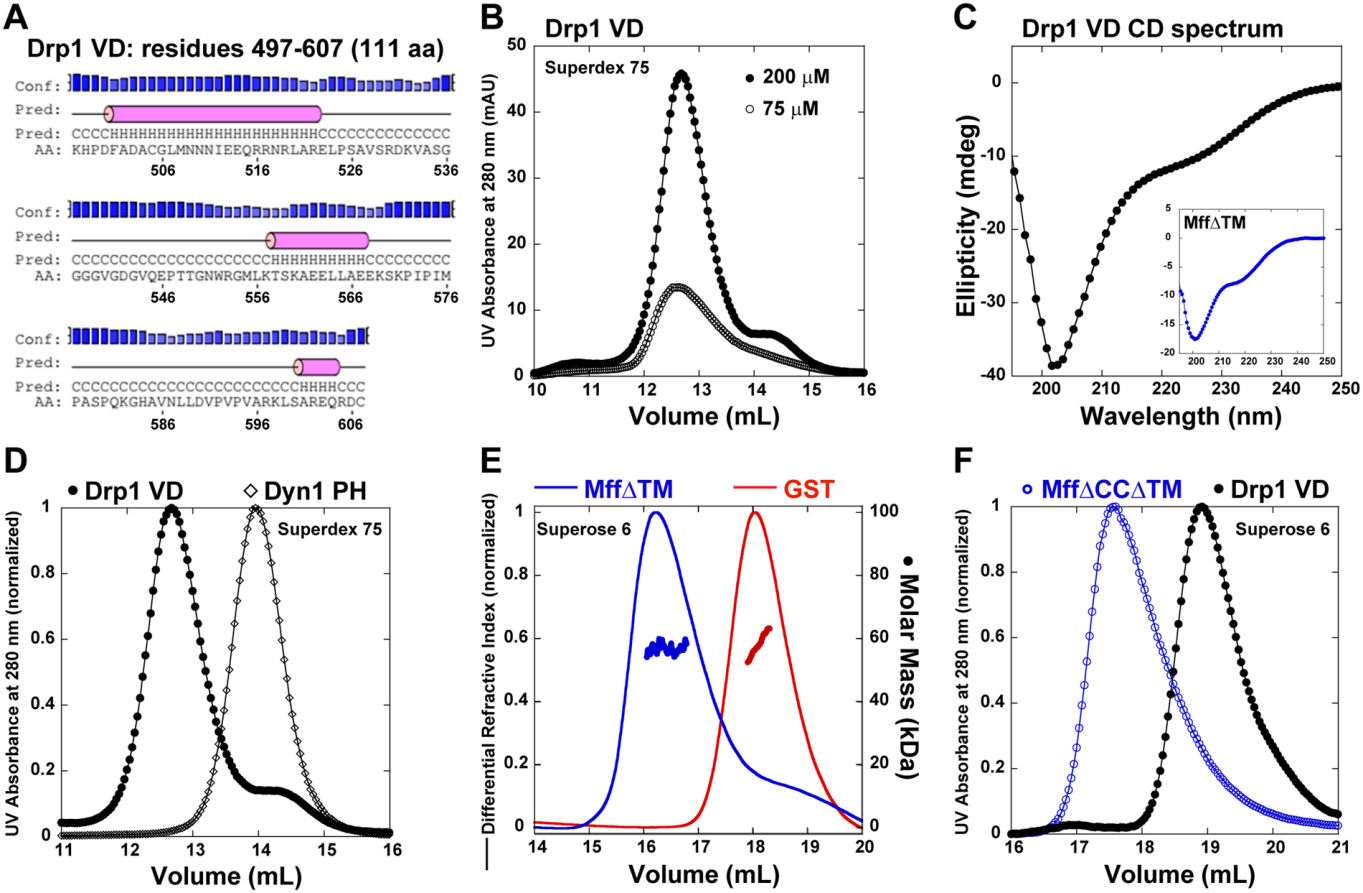
Drp1 VD is monomeric and does not directly participate in self-assembly. (**A**) Secondary structure prediction of the Drp1 VD sequence determined by PSIPRED^84^. (**B**) SEC elution profiles of GST-excised, tag-free Drp1 VD loaded at 75 and 200 μM onto a Superdex 75 10/300 GL column. (**C**) CD spectrum of 200 μM 6X His-tagged Drp1 VD. *Inset*, CD spectrum of 17.2 μM MffΔTM is shown for comparison. (**D**) SEC elution profiles of tag-free Drp1 VD (200 μM) and Dyn1 PH domain (40 μM; ~14 kDa monomer) loaded onto a Superdex 75 10/300 GL column. (**E**) SEC-MALS profiles of dimeric MffΔTM (~27 kDa monomer × 2) and dimeric GST (~25 kDa monomer × 2) loaded onto a Superose 6 10/300 GL column. (**F**) SEC elution profile of monomeric MffΔTMΔCC (~22 kDa)^36^ relative to Drp1 VD, loaded at 75 and 45 μM, respectively, onto a Superose 6 10/300 GL column.

We determined that the VD is unlikely to participate directly in higher-order Drp1 self-assembly. In contrast to the isolated dynamin PH domain that exists as a monodisperse monomer in solution^28^, the isolated Drp1 VD was previously characterized as a homotetramer based on SEC analysis against known, globular protein size standards^59^. Prediction algorithms however indicate that the Drp1 VD, unlike the globular dynamin PH domain, is an IDR and likely exists in an extended conformation (Fig. 4A). Protein shape influences SEC and most elongated proteins (IDRs) anomalously elute at a faster rate than globular proteins of comparable size^60–62^. This renders any estimation of size based on elution profile comparison of disparately shaped proteins unreliable.

We therefore sought to determine the oligomeric state of the Drp1 VD unambiguously. Despite being intrinsically disordered, the Drp1 VD purifies as a highly soluble protein and remains stable at high micromolar concentrations (up to at least 0.5 mM) consistent with previous findings^59^. SEC elution analyses of Drp1 VD at two different loading concentrations revealed a similar peak profile indicating the presence of a monodisperse molecular population and the absence of dynamic oligomerization equilibria (Fig. 4B). Circular dichroism (CD) spectroscopy ascertained that the Drp1 VD is largely unstructured in solution (Fig. 4C). The far-UV spectrum of Drp1 VD showed a minimum at 202 nm and a shoulder at 222 nm consistent with a predominantly random-coil structure with minimal helical content. The Drp1 VD CD spectrum was comparable to the CD spectrum of mitochondrial fission factor (MffΔTM), an essential Drp1 adaptor at the mitochondrial surface^11^, also predicted to be largely unstructured (Fig. 4C, *inset*). However, the lack of sufficient light scattering from the disordered Drp1 VD even at a highest loading concentration (≥200 μM) precluded SEC-MALS determination of its oligomeric state. We therefore resorted to conventional SEC, this time however, by comparing the Drp1 VD to proteins of comparable shape and/or size.

Much in keeping with an elongated shape and/or a higher oligomeric state, the Drp1 VD (~12 kDa monomer) eluted faster than the comparably sized, but globular, dynamin PH domain (~14 kDa monomer) (Fig. 4D). Also consistent with the influence of protein shape on SEC elution, the similarly unstructured (Fig. 4C, inset), but dimeric, MffΔTM (~27 kDa monomer × 2) eluted faster than the comparably sized, but globular, glutathione S-transferase dimer (GST; ~27 kDa monomer × 2) (Fig. 4E). We therefore utilized Mff as a shape-appropriate IDR size marker for Drp1ΔVD. A direct comparison of Drp1 VD elution to that of a monomeric MffΔTM variant, MffΔCCΔTM (~22 kDa), revealed that the Drp1 VD is a monomer (Fig. 4F). Thus, the monomeric Drp1 VD, similar to the dynamin PH domain, likely does not participate directly in higher-order Drp1 self-assembly.

### An intramolecular VD interaction site sterically controls G-domain dimerization

Drp1ΔVD polymers exhibit a significantly lower cooperative GTPase activity compared to Drp1 WT, both in solution and on membranes^31,48^. Drp1ΔVD polymers also do not tubulate membranes owing to a pronounced defect in helical self-assembly^16,48^. These data indicate that the stalk-driven self-assembly of Drp1 in the absence of the VD significantly impairs nucleotide-dependent, inter-rung G-domain dimerization.

Previous studies, however, have shown that Mff binds Drp1ΔVD more avidly than full-length Drp1 to nucleate higher-order Drp1-Mff co-assembly resulting in a more robust stimulation of cooperative Drp1 GTPase activity^36^. These data suggested that a conformational rearrangement of the VD induced by Mff- and/or membrane-interactions, mimicked here by the Drp1ΔVD, increases or optimizes Drp1 G-domain dimerization for greater cooperative GTPase activity.

**Figure 5:**
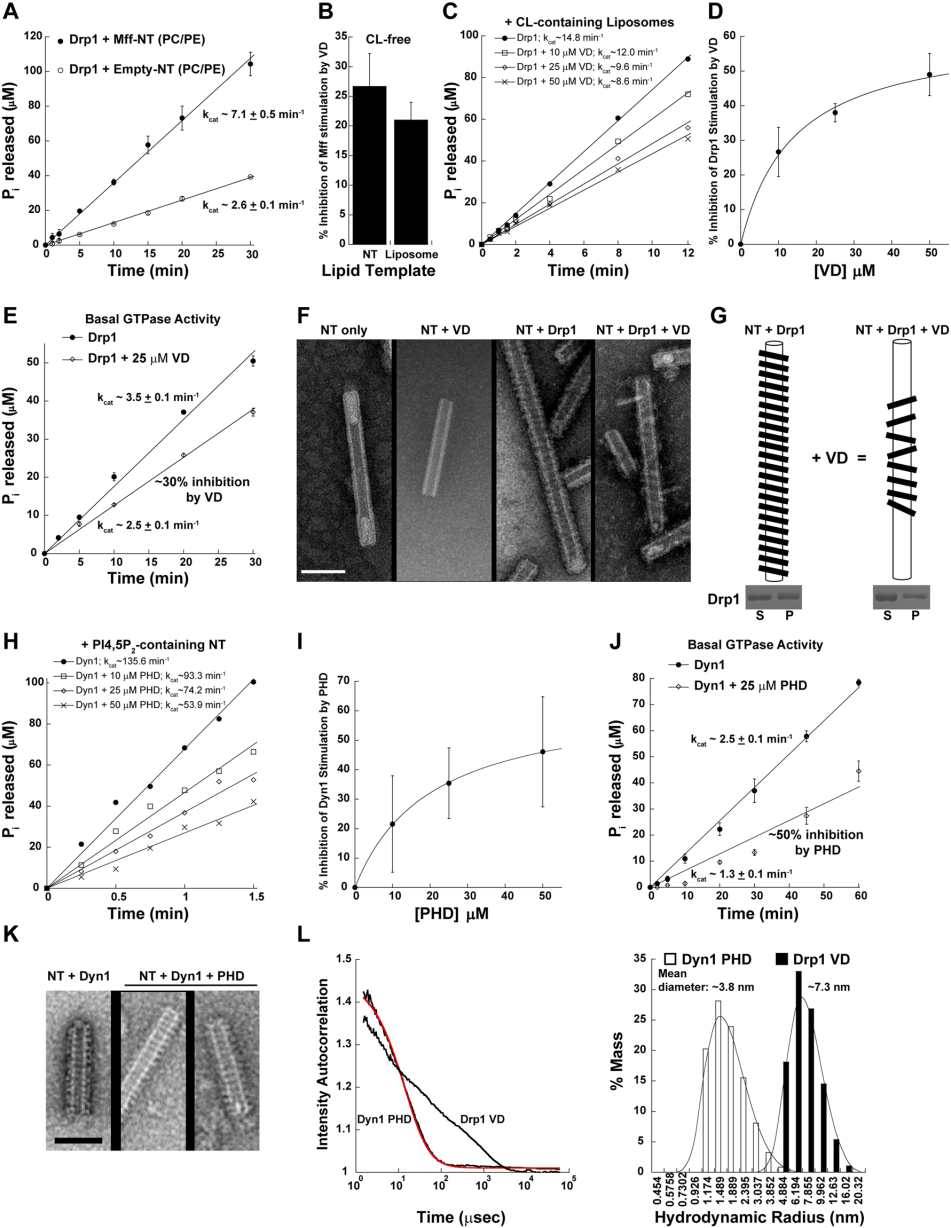
Evidence for an intramolecular VD interaction site that controls G-domain dimerization. (**A**) Stimulated Drp1 GTPase activity on Mff-reconstituted CL-free NT relative to empty NT. (**B**) % inhibition of Mff-stimulated Drp1 GTPase activity on CL-free NT and liposomes in the presence of a vast molar excess (25 μM) of 6X His-tagged Drp1 VD. (**C**) Inhibition of CL-stimulated Drp1 GTPase activity on liposomes in the presence of increasing concentrations of 6X His-tagged VD. (**D**) % inhibition of CL-stimulated Drp1 GTPase activity on liposomes as a function of 6X His-tagged VD concentration. (**E**) Basal Drp1 GTPase activity in the absence and presence of 25 μM 6X His-tagged VD. (**F**) Representative EM images of 25 mol% CL-containing NT in the absence and presence of Drp1 and/or isolated VD. Scale bar, 100 nm. (**G**) Graphical illustration of the isolated VD-induced morphological changes in the Drp1 helical polymer. *Inset* below shows S and P fractions of Drp1 WT incubated with 25 mol% CL-NT in the absence (*left panel*) and presence of a vast molar excess of isolated Drp1 VD (*right panel*) obtained by high-speed centrifugation and visualized by SDS-PAGE and Coomassie staining. (**H**) Inhibition of PIP_2_-stimulated Dyn1 GTPase activity on NT in the presence of increasing concentrations of the isolated Dyn1 PH domain. (**I**) % inhibition of PIP_2_-stimulated Dyn1 GTPase activity on NT as a function of isolated Dyn1 PH domain concentration. (**J**) Basal Dyn1 GTPase activity in the absence and presence of 25 μM isolated Dyn1 PH domain. (**K**) Representative EM images of 10 mol% PIP_2_-containing NT incubated with Dyn1 in the absence and presence of a vast molar excess of the isolated Dyn1 PH domain. Scale bar, 100 nm. (**L**) (*left panel*) DLS intensity autocorrelation curves for the isolated, untagged Dyn1 PH domain (PHD) and the isolated, untagged Drp1 VD. Data for the Dyn1 PH domain was best fit (red trace) to a monodisperse, monomeric population. Drp1 VD data exhibited polydispersity indicative of an ensemble of conformational states for the monomeric domain. *(right panel)* Distribution of the hydrodynamic radii calculated from DLS measurements for the isolated Dyn1 PHD and the Drp1 VD. The mean diameter of each domain is indicated above.

To explore the existence of such a Drp1 VD conformational rearrangement, we reconstituted full-length Mff in proteo-lipid nanotubes (Mff-NT) composed of the major mitochondrial outer membrane (MOM) phospholipids, phosphatidylcholine (PC) and phosphatidylethanolamine (PE)^35^. Uniformly cylindrical NT of ~30-nm diameter, previously utilized by us^35,48^, was chosen as a biomimetic template of pre-constricted ER-mitochondria contact sites that harbor Mff and localize Drp1 self-assembly for mitochondrial fission^63^. In the absence of Mff, zwitterionic PC/PE-containing liposomes or NT does not recruit Drp1^15,35,48^. As previously shown, we observed a ~3-fold increase in the cooperative GTPase activity of Drp1 in the presence of Mff-NT compared to empty-NT^15^ (Fig. 5A). However, in the presence of a vast molar excess of the isolated Drp1 VD in the reaction mixture, the Mff-stimulation of Drp1 GTPase activity was significantly diminished (~25%) (Fig. 5B). Mff-proteoliposomes yielded similar results indicating that the VD inhibition of Mff-induced Drp1 GTPase stimulation occurs independently of membrane curvature (Fig. 5B). Importantly, these data indicated the presence of an exposed, intramolecular VD interaction site in full-length Drp1 that perturbs functional Drp1-Mff interactions. As VD inhibition of Mff-stimulated Drp1 GTPase activity occurred independently of Drp1-CL interactions, these data suggest that a conformational rearrangement of the Drp1 VD elicited by upstream Mff interactions primes nucleotide-dependent G-domain dimerization for cooperative GTPase activity.

Structural and functional data have shown that the PH domain of dynamin resides in two different orientations, ‘closed’ and ‘open’ conformations, relative to the stalk^22,24^. In solution (cytosol), the dynamin PH domain interfaces with the stalk (closed conformation) to sterically auto-inhibit premature dynamin self-assembly and restrict cooperative GTPase activity. Upon encountering a PIP_2_-containing target membrane, the PH domain is displaced from this stalk interface (open conformation) to mediate specific phospholipid interactions and promote helical self-assembly. We likened a similar scenario for Drp1, wherein the VD is displaced from a putative intramolecular interaction site or interface upon specific Mff interactions. Under our experimental conditions, we reasoned that the presence of isolated VD in vast molar excess in solution reoccupies the interface vacated by the covalently linked VD, resulting in a re-auto-inhibition of Drp1 self-interactions and cooperative GTPase activity.

To test this hypothesis and determine the structural basis of VD-mediated Drp1 auto-inhibition, we used CL-containing NT that favors ordered helical Drp1 self-assembly and robustly stimulates Drp1 cooperative GTPase activity compared to Mff-NT^15,35^. Titration of isolated VD in a mixture comprised of full-length Drp1 and CL-containing NT similarly resulted in a robust concentration-dependent VD inhibition of Drp1 cooperative GTPase activity (Fig. 5C,D). Remarkably, the isolated VD similarly auto-inhibited Drp1 basal GTPase activity in solution, in the absence of Mff and CL, indicating that the intrinsic VD reversibly interacts with this intramolecular interaction site and provides dynamic access to the isolated VD, which then robustly suppresses Drp1 cooperative basal GTPase activity.

Negative-stain EM analysis of NT-decorated full-length Drp1 in the absence and presence of the isolated Drp1 VD revealed the structural basis of VD-mediated Drp1 auto-inhibition (Fig. 5F). Like dynamin^43^, Drp1 forms highly ordered helical polymers on CL-containing NT even in the absence of nucleotide^35^ (Fig. 5F). Juxtaposition of adjacent helical rungs promotes nucleotide-dependent inter-rung Drp1 G-domain dimerization and cooperative GTPase activity^16,43,64^. Remarkably, in the presence of a vast molar excess of VD, Drp1 still constituted helical polymers, indicating that the reoccupation of the putative intramolecular interface by the isolated VD does not impair stalk-mediated Drp1 self-assembly (Fig. 5F). However, these Drp1 helices displayed a relaxed or distended conformation, akin to that described for Dyn1 in the presence of GDP previously^43^ (Fig. 5F,G). Moreover, the helices were visibly impaired in inter-rung Drp1 self-interactions that primarily involve G-domain dimerization^18^. Critically, Drp1 binding to CL-containing NT was not significantly affected in the presence of a vast molar excess of isolated VD, as determined by a spin-sedimentation assay^35^, indicating that the intrinsic and isolated VDs do not compete for membrane binding under these conditions (Fig. 5G (bottom panel), Supplementary Fig. S4). Collectively, these data indicated that the auto-inhibitory intramolecular interactions of the VD sterically interfere with helical inter-rung G-domain dimerization, and as a result, diminish cooperative GTPase activity.

We next explored whether a similar mechanism also operates in Dyn1, which likewise contains a flexibly tethered PH domain that samples multiple orientations relative to the stalk as evidenced in the recently available crystal structures^22–24^. Titration of the isolated Dyn1 PH domain, not surprisingly, resulted in a similar concentration-dependent inhibition of Dyn1 cooperative GTPase activity, both in solution and on PIP_2_-containing membranes (Fig. 5H–J). However, unlike in the case of Drp1, no conspicuous difference in the geometry of the helical Dyn1 polymers was observed even in the presence of a vast molar excess of the isolated PH domain, indicating a difference likely in the respective hydrodynamic volumes of the isolated Dyn1 PH domain and the Drp1 VD (Fig. 5K). Indeed, dynamic light scattering (DLS) measurements revealed that the globular Dyn1 PH domain (125 aa) is monodisperse and exhibits a hydrodynamic radius of ~1.9 nm consistent with its crystal structure^65^ (Fig. 5L). However, the monomeric Drp1 VD of a similar polypeptide length (111 aa) appeared polydisperse, as indicated by a broad intensity auto-correlation trace, which was unable to be fit to a size model unlike for the Dyn1 PH domain (Fig. 5L, left panel). These data suggest that the Drp1 VD samples an ensemble of conformations ranging presumably from the very compact to largely distended states with an average hydrodynamic radius of ~3.7 nm (Fig. 5L, right panel). Based on these results, we suggest that the greater hydrodynamic volume as well as the robust conformational dynamics of the Drp1 VD relative to the Dyn1 PH domain, likely manifests as a loosely packed Drp1 helical polymer with an enhanced inter-subunit spacing relative to Dyn1, as evidenced recently by cryo-EM^18^. Nevertheless, these studies establish that Drp1 and Dyn1, although structurally disparate in their membrane-interaction regions, are mechanistically conserved.

### The Drp1 VD is necessary and sufficient for Drp1-phospholipid interactions

We also addressed the question of whether the Drp1 VD, displaced from the putative intramolecular interface, directly effects specific phospholipid interactions. Although Drp1ΔVD has been shown to bind negatively charged membranes via electrostatic interactions mediated by the membrane-proximal base of the stalk^31,32,48^, the specificity and functional contribution of this interaction remain uncertain. Point mutations in the VD (e.g., the Drp1 4KA quadruple mutant described below) previously identified to impair specific Drp1-phospholipid interactions have since been found paradoxically to also impair Drp1 self-assembly^30,48^. Whether stalk-driven Drp1 self-assembly is essential for stable Drp1-phospholipid interactions therefore remains unclear.

**Figure 6:**
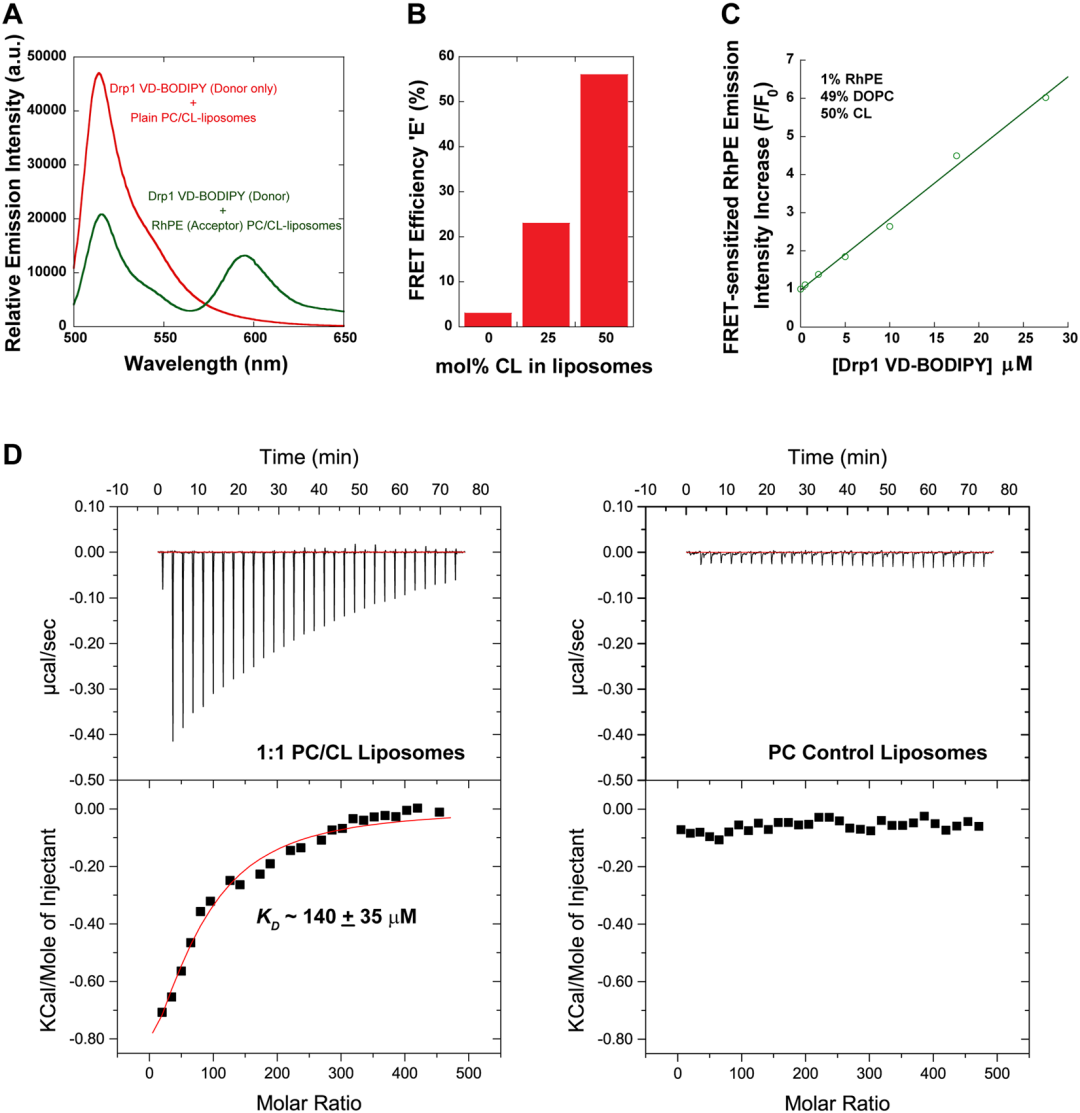
Drp1 VD is necessary and sufficient for specific CL interactions. (**A**) Representative emission spectra for BODIPY-FL (donor)-labeled 6X His-tagged Drp1 VD (27.5 μM final) in the absence and presence of RhPE (acceptor; 1 mol%) in 50 mol% CL-containing liposomes (2.5 mM total lipid final). FRET was determined by the decrease in donor emission intensity at 510 nm concomitant with the FRET-sensitized increase in acceptor emission intensity at 590 nm upon donor excitation at 470 nm. The spectra were corrected for background as well as for the direct excitation of the acceptor at the donor excitation wavelength. (**B**) FRET efficiency, E, was determined as described under Methods for BODIPY-FL-labeled Drp1 VD (27.5 μM final) as a function of increasing CL concentration in RhPE-labeled liposomes (2.5 mM total lipid final). (**C**) FRET-sensitized increase in RhPE (acceptor) emission intensity is plotted as a function of increasing BODIPY-FL (donor)-labeled 6X His-tagged Drp1 VD concentration. F_0_ is the initial intensity of RhPE at 590 nm in the absence of donor, and F is the final intensity of RhPE at 590 nm upon incubation with donor for 30 min at room temperature. Representative FRET data are shown. (**D**) ITC equilibrium binding isotherm for Drp1 WT (2 μM) titrated with 50 mol% CL-containing liposomes (left), or with control 100% DOPC liposomes (right), at 25 °C. Each downward spike represents a single injection of liposomes into the sample cell. The binding constant *K*_*D*_ in the case of 50 mol% CL-containing liposomes was determined by fitting the area under the spikes to a binding isotherm.

To resolve this paradox, we examined the membrane binding properties of the isolated Drp1 VD, which serves as a mimetic of the displaced VD in full-length Drp1. As previously utilized for full-length Drp1^48^, we monitored Förster resonance energy transfer (FRET) between BODIPY-Fl (donor)-labeled Drp1 VD and rhodamine-PE (RhPE; acceptor)-labeled target membranes. A high magnitude of FRET was detected between Drp1 VD-BODIPY and RhPE-labeled CL-containing liposomes measured spectrally by the substantial reduction in donor emission intensity and the concomitant increase in sensitized acceptor emission intensity upon donor excitation (Fig. 6A). Titration experiments revealed very little FRET for CL-free, control liposomes in contrast to substantial increases in FRET efficiency for liposomes with increasing CL content (up to 50 mol%) (Fig. 6B). These data indicated that Drp1 VD is sufficient for specific CL interactions. Furthermore, Drp1 VD titration at a constant liposome concentration revealed a linear (non-sigmoidal) increase in FRET-sensitized acceptor emission intensity (Fig. 6C). These data indicated that the Drp1 VD, which is monomeric in solution, does not bind CL cooperatively on membranes. We utilized label-free isothermal titration calorimetry (ITC) to determine the *K*_*D*_ (equilibrium dissociation constant) of Drp1 VD-CL association (Fig. 6D). Liposome titration at a constant Drp1 VD concentration revealed only a weak interaction between the isolated Drp1 VD and 50 mol% CL-containing liposomes (*K*_*D*_ ~140 ± 35 μM for total lipid; ~70 ± 18 μM for CL). Consistent with a CL-specific interaction, Drp1 VD did not bind control PC liposomes. Nevertheless, these data reveal that in the absence of stalk-mediated Drp1 self-assembly and consequent multivalent membrane interactions, the isolated Drp1 VD requires a greater than physiological concentration of CL (>10 mol%)^66^ for stable membrane association.

We previously showed that the VD in full-length Drp1 functions primarily to cluster CL underneath the growing Drp1 helical scaffold on membranes^48^. Our data with the isolated VD therefore suggests that CL clustered to high local concentrations underneath the growing Drp1 scaffold ultimately stabilizes VD-membrane interactions. Thus, it appears, as previously determined for the dynamin PH domain^25,36,47^, the Drp1 VD utilizes high avidity multivalent interactions, instead of individual high affinity interactions, to enable stable membrane association. The *K*_*D*_ values obtained here for isolated Drp1 VD-CL interactions at 50 mol% CL, closely approximate the *K*_*D*_ values obtained for isolated Dyn1 PH domain-PIP_2_ interactions at only 3 mol% PIP_2_ assuming a 1:1 PH domain:PIP_2_ stoichiometry^25^. These data therefore suggest that each Drp1 VD monomer binds multiple CL molecules at greater than a 1:1 stoichiometry, an assertion consistent with its unstructured, extended conformation and our previous results^48^.

### VD rearrangement necessitates basal GTP hydrolysis-driven Drp1 polymer disassembly

To determine the molecular requirements for stable Drp1 VD-membrane association, we reassessed the biochemical and biophysical properties of two Drp1 mutants, Drp1 4KA and Drp1 R247 A, previously shown to be impaired in direct CL binding^30,67^. Drp1 4KA is a quadruple mutant of the VD, wherein four closely spaced Lys (K) residues are substituted with Ala (A)^30^, whereas Drp1 R247A contains a single amino acid residue substitution, puzzlingly, in the GTPase domain far away from the membrane surface^18,67^.

**Figure 7:**
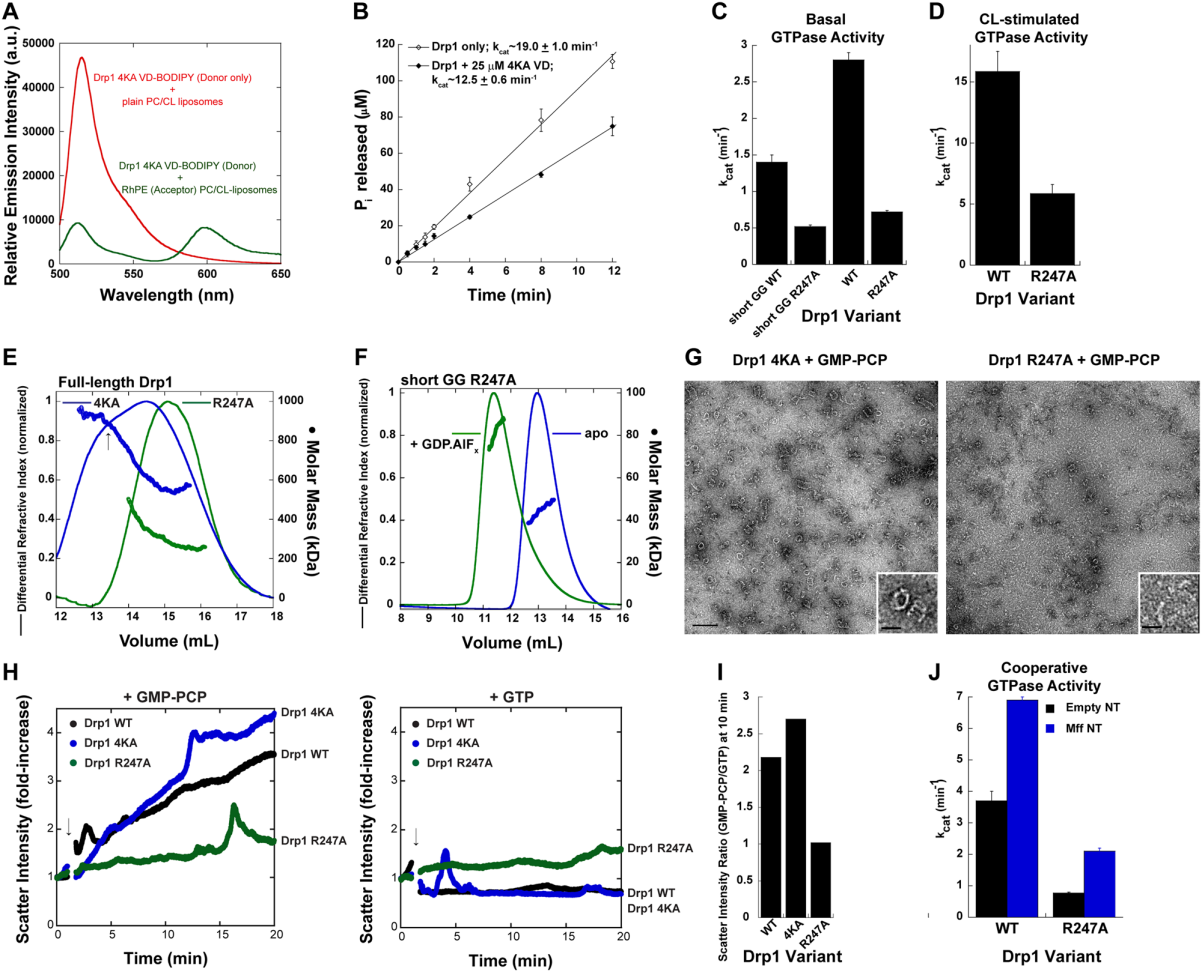
Basal GTP hydrolysis generates Drp1 dimers from cytosolic oligomers for mitochondrial recruitment. (**A**) Same as Fig. 6A but with BODIPY-FL-labeled 6X His-tagged Drp1 4KA VD. (**B**) Inhibition of CL-stimulated Drp1 GTPase activity on liposomes in the presence of a molar excess of 6X His-tagged 4KA VD. (**C**) Basal GTPase activities of the Drp1 R247A mutant in the short GG and full-length backgrounds relative to Drp1 WT. (**D**) Lipid-stimulated GTPase activity of Drp1 R247A relative to Drp1 WT on 25 mol% CL-containing liposomes. (**E**) SEC-MALS profiles of full-length Drp1 4KA and Drp1 R247A loaded at 10 μM onto a Superose 6 10/300 GL column. (**F**) SEC-MALS profiles of Drp1 short GG R247A loaded at 25 μM onto a Superdex 75 10/300 GL column in the absence of, and upon incubation with, GDP.AlF_x_. (**G**) Representative EM images of Drp1 4KA (*left panel*) and Drp1 R247A (*right panel*) incubated in the presence of GMP-PCP. Scale bar, 200 nm. *Insets* show magnified images of the oligomers. *Inset* scale bar, 50 nm. (**H**) Time-dependent 90° light scattering intensity changes for Drp1 4KA and Drp1 R247A relative to Drp1 WT upon addition of either GMP-PCP (left panel) or GTP (right panel) as indicated by arrows. The data are normalized to the initial scatter value prior to nucleotide addition to determine the fold-change in scatter intensity upon nucleotide addition. (**I**) Ratio of 90° scatter intensities for Drp1 WT and mutants in the presence of GMP-PCP, which stimulates Drp1 oligomer self-assembly, over the presence of GTP, which stimulates oligomer disassembly, determined after 10 min of incubation with nucleotide (from panel H). The higher the ratio, the greater is the propensity for higher-order self-assembly. Drp1 R247A with nearly identical scatter values in the presence of both GMP-PCP and GTP (ratio of ~1) therefore exhibits defects in both self-assembly and disassembly. (**J**) Mff-stimulated GTPase activity on NT of Drp1 R247A relative to Drp1 WT. Empty NT serves as the control for baseline activity.

Unexpectedly, we detected a similar magnitude of FRET between the isolated Drp1 4KA VD and CL-containing liposomes as for wild-type Drp1 VD (Fig. 7A). These data indicated that the four K residues are likely not involved in direct Drp1-CL interactions. Likewise, titration of isolated Drp1 VD 4KA into a reaction mixture containing full-length Drp1 WT and CL-containing liposomes elicited a similar reduction in Drp1 GTPase activity as with Drp1 VD WT, suggesting that these K residues also do not participate in auto-inhibitory, intramolecular VD interactions (Fig. 7B).

Likewise, contradictory to a previous report^67^, we found that Drp1 R247A was instead defective in both basal (~3-fold reduction in activity in both short GG and full-length Drp1 backgrounds) and lipid-stimulated cooperative GTPase activities relative to Drp1 WT *in vitro* (Fig. 7C,D), akin to that reported for Drp1 4KA previously^25,41^. Nevertheless, Drp1 4KA and Drp1 R247A, however, are both impaired in mitochondrial fission *in vivo*^48,67^.

To more accurately determine the mechanistic roles of these residues, we first determined the previously unexplored oligomerization propensities of Drp1 4KA and Drp1 R247A (Fig. 7E) relative to Drp1 WT in solution (Fig. 2A). SEC-MALS analyses revealed that Drp1 4KA favored the formation of predominantly higher-order oligomers in solution versus mostly dimers and tetramers favored by both Drp1 R247A and Drp1 WT under these conditions (Figs 2A and 7E). As dimers, and not higher-order oligomers, potentiate Drp1 helical self-assembly and cooperative GTPase activity on CL-containing membranes^35^, these data suggested that the markedly enhanced oligomeric propensity of Drp1 4KA in solution, rather than a specific impairment in direct phospholipid interactions, perturbs Drp1 4KA association and higher-order self-assembly on membranes. Conversely, given the location of the mutation within a nucleotide-binding structural element (G5 motif) of the GTPase domain^17^, we reasoned that a broader defect in the capacity of Drp1 R247A to undergo dynamic cycles of assembly and disassembly upon GTP binding and hydrolysis in solution, respectively, impairs its capacity to generate assembly-competent dimers for higher-order self-assembly on membranes. SEC-MALS analysis of Drp1 short GG R247A revealed that the mutation does not impair transition state-dependent G-domain dimerization indicating that a lack of GTP binding itself is not the primary defect (Fig. 7F).

Consistent with our notion, negative-stain EM visualization of Drp1 4KA and Drp1 R247A revealed that whereas Drp1 4KA formed higher-order helical oligomers in solution in relative abundance in the presence of GMP-PCP, Drp1 R247A self-assembly was more limited to the formation of smaller arcs and aggregates (Fig. 7G). We used 90° light scattering to quantify the relative capacities of Drp1 WT, Drp1 4KA, and Drp1 R247A to form higher-order oligomers upon GTP (GMP-PCP) binding and undergo disassembly upon GTP hydrolysis (Fig. 7H). As expected, GMP-PCP binding was accompanied by a robust increase in light scattering intensity for both Drp1 WT and Drp1 4KA (Fig. 7H, *left panel*), with Drp1 4KA displaying a greater propensity for oligomer formation, consistent with the favored higher-order oligomerization state of this mutant as indicated by SEC-MALS (Fig. 7E). On the other hand, GMP-PCP addition elicited only a modest increase in Drp1 R247A (Fig. 7H, *left panel*), consistent with a severely impaired propensity to form oligomers upon GTP binding (Fig. 7I). Conversely, GTP addition (GTP hydrolysis) elicited no increase in light scattering intensity for either Drp1 WT or Drp1 4KA, indicative of a molecular population undergoing steady state cycles of GTP binding-dependent assembly and GTP hydrolysis-dependent disassembly (Fig. 7H, *right panel*). On the other hand, Drp1 R247A showed a slow but steady increase in light scattering intensity even in the presence of GTP, as with GMP-PCP. This indicated a markedly reduced capacity of the newly formed oligomers to disassemble upon GTP hydrolysis, also consistent with an impaired basal GTPase activity (Figs 7H,I).

**Figure 8:**
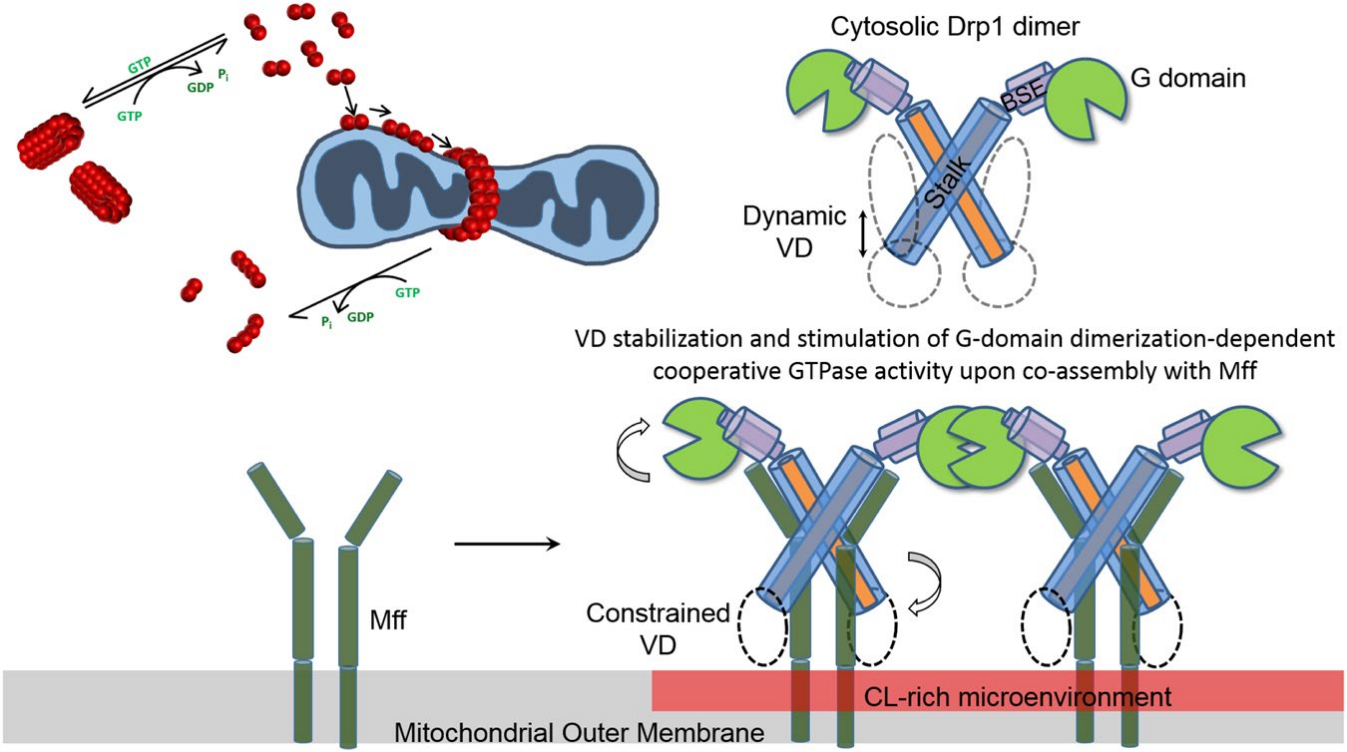
Model for hierarchical Mff-and CL-induced Drp1 conformational rearrangements during mitochondrial fission. (Top left) Model of Drp1 dimer nucleation of higher-order Drp1 self-assembly on the mitochondrial surface. (Top right and bottom) Cartoon illustration of the putative, domain-specific conformational rearrangements in the mitochondrial outer membrane-bound Drp1 dimer that stabilize Drp1-Mff interactions and optimize nucleotide-dependent G-domain dimerization for robust cooperative GTPase activity. Initial Drp1 dimer binding to Mff constrains VD dynamics and effectively displaces the VD from the intramolecular interaction site. This subsequently facilitates VD sampling of the membrane microenvironment for the presence of target phospholipids. Specific CL interactions anchors the VD onto the membrane surface, and alleviates the strong VD-mediated steric interference of helical inter-rung G-domain dimerization, which results in a greater cooperative GTPase activity geared toward membrane constriction and fission.

As Drp1 dimers, and not oligomers, are recruited by Mff to nucleate Drp1-Mff co-assembly for stimulated Drp1 cooperative GTPase activity^36^, we tested whether Mff could activate Drp1 R247A similar to Drp1 WT. Consistent with the reduced generation of solution dimers owing to an impaired basal GTP hydrolysis, Drp1 R247A exhibited a similar 3-fold reduction in Mff-stimulated GTPase activity compared to Drp1 WT (Fig. 7J). Regardless, the fold-increase in Mff stimulation of Drp1 GTPase activity over the corresponding basal GTPase activity was quantitatively similar between Drp1 WT and Drp1 R247A. These data indicated that the diminished oligomer disassembly and dimer formation in solution for Drp1 R247A, quantitatively impairs Drp1 R247A dimer recruitment to target membranes. We therefore conclude that Mff-directed VD conformational rearrangement, which is essential for the alleviation of VD-mediated Drp1 auto-inhibition, is contingent upon the basal GTP hydrolysis-dependent generation of Drp1 dimers from oligomers in solution (Fig. 8). The Drp1 basal GTPase activity in the cytosol thus appears to function as a critical regulator of Drp1 mitochondrial recruitment for fission.

## Discussion

We here define common operational principles, as well as identify various unique design features that relate the structurally disparate DRPs to the classical dynamins. Using the human Drp1 as an archetype, we demonstrate that nucleotide-dependent G-domain dimerization, independent of stalk-mediated higher-order self-assembly, is the major driver of cooperative GTP hydrolysis in both Drp1 and Dyn1, both in solution and on membranes. In the physiological context, assembly-independent G-domain dimerization likely translates to the basal GTPase activity previously described for dynamin, which functions as a ‘kinetic monitor’ of endocytic pit maturation prior to curvature-driven self-assembly and membrane fission^68–70^, and to the regulation of Drp1 dimer availability in the cytosol for subsequent mitochondrial recruitment and self-assembly for fission as demonstrated here (Fig. 8). Drp1 G350D, which exhibits maximal cooperative GTPase activity despite the absence of helical self-assembly, corroborates our notion that cooperative GTP hydrolysis in the unassembled state likely originates from nucleotide-dependent G-domain dimerization interactions facilitated by collisional encounters between Drp1/Dyn1 dimeric or tetrameric subunits, either in solution or on membranes prior to helical higher-order self-assembly.

We show that an IDR insert unique to the Drp1 GTPase domain, the 80-loop, functions akin to the unstructured Dyn1 PRD, both as a positive regulator of G-domain dimerization on its own, and as a negative regulator of G-domain dimerization and cooperative GTPase activity in the presence of the A-insert (the extended 80-loop). Consistently, the Dyn1 PRD was previously shown to positively regulate Dyn1 self-assembly^40,41^, while also simultaneously to negatively regulate cooperative GTP hydrolysis^34^. We show here that the PRD-imposed negative regulation of Dyn1 cooperative GTPase activity also occurs independent of stalk-mediated higher-order self-assembly, albeit via disparate mechanisms to Drp1. Although no crystal structures of Drp1 long GG or of Dyn1 GG-PRD exist yet, we speculate that the A-insert-extended Drp1 80-loop suppresses G-domain dimerization and cooperative GTP hydrolysis via steric interference. The A-insert-free 80-loop, on the other hand, functions to cross-link partnering GTPase domains to promote cooperative GTP hydrolysis, as G-domain dimerization appears to be impaired in its absence. Likewise, we predict that the Dyn1 PRD also functions to cross-link partnering GTPase domains, prevent G-domain dimer dissociation post-GTP hydrolysis, and suppress rapid progression of the GTPase domains through multiple rounds of the GTP hydrolysis cycle, resulting in a lower GTP hydrolysis rate. Thus, we propose that the Drp1 80-loop and the Dyn1 PRD both function as ‘kinetic timers’ of cooperative GTP hydrolysis.

Based on our results, we further predict that the various protein partners that bind dynamin via SH3 domain-PRD interactions (e.g. SNX9) and stimulate cooperative GTPase activity, do so via the alleviation of the PRD-effected auto-inhibition of cooperative GTP hydrolysis^33,49,71^. Whereas similar partner protein binding interactions for the extended Drp1 80-loop have been proposed based on homology to dynamin A (DymA) from *Dictyostelium discodeum* that also contains an extended IDR, identified as a potential protein-protein interaction site, at a comparable position, this remains to be fully explored. However, consistent with such a role, Drp1 isoforms that undergo alternatively splicing in the 80-loop region are differentially stimulated by Mff^15^. Whether Mff or other binding partners directly interact with this region or promote G-domain dimerization and cooperative GTPase activity through other allosteric mechanisms remains to be determined. A role for 80-loop-promoted intersubunit G-domain dimerization in the stable recruitment of Drp1 to mitochondria however reconciles conflicting data concerning the preferred oligomerization state of cytosolic Drp1 recruited by the dominant adaptor, Mff, at the mitochondrial surface^36,72^. We suggest that Mff, which preferentially recruits minimal Drp1 dimers from the cytosol, functions to stabilize Drp1 on the mitochondrial surface by effecting inter-dimer G-domain dimerization (*trans* tetramerization). Thus, Mff stimulates Drp1 cooperative GTPase activity via the nucleation of higher-order Drp1 self-assembly^15,36^.

We establish a key mechanistic role for the conserved L1N loop of the stalk in the regulation of Drp1 helical self-assembly and cooperative GTPase activity. Bounded by identically positioned Gly residues in both dynamin (G346 and G359) and Drp1 (G350 and G363)^16,22–24^, the L1N loop also contains a central stretch of highly conserved residues, ^351^IDTYE^355^ in Dyn1 and ^355^IETSE^359^ in Drp1, both of which carry a net negative charge. Remarkably, in the recently solved crystal structure of the dynamin tetramer^24^, the L1N loop of each dynamin monomer in the dimeric repeating subunit interacts with the positively charged self-assembly interface 3 of another monomer in the adjacent dimer. This electrostatic interaction is predicted to position the PH domain in an auto-inhibitory conformation in solution, as well as set the register (curvature and pitch) of dynamin helical self-assembly on membranes by orienting the repeating dimeric subunits of the polymer at a precise angle relative to one another^17,19^. A similar mechanism presumably operates in Drp1 as the G350D L1N mutation, which likely augments such electrostatic interactions, retains Drp1 as a VD auto-inhibited, helical self-assembly-defective, minimal dimer in solution. Nevertheless, Drp1 G350D dimers cooperatively hydrolyze GTP at a significantly faster rate than Drp1 WT. Interestingly, a corresponding enhancement of basal GTPase activity is also observed for Dyn1 stabilized in the auto-inhibitory closed conformation through an engineered cross-link between Y354C in the L1N loop and native C607 in the PH domain^54^. These parallel observations between Drp1 and Dyn1 lend support to our notion that the stalk mutation G350D stabilizes Drp1 stalk-VD interactions and helps retain the Drp1 VD in an auto-inhibitory closed conformation. Importantly, these data further establish that G-domain dimerization and stimulated GTPase activity are not dependent on helical Drp1 or dynamin self-assembly. Our data instead reveal that the narrower helical geometry of Drp1 self-assembly in solution (~50 nm diameter), versus on membranes (typically greater than 100 nm)^35^, functions primarily to restrict rather than to promote G-domain dimerization for maximal cooperative GTPase activity.

Removal of the second IDR, the VD, by contrast to that of the 80-loop, results in the unrestrained self-assembly of Drp1 (Drp1ΔVD) in solution. In contrast to Drp1 WT that constitutes polymers of helical geometry, Drp1ΔVD forms filamentous polymers in solution that self-organize further to form laterally stacked filamentous bundles. These data indicate a secondary role for the VD in the self-assembly of Drp1 in the proper helical register. Drp1ΔVD polymers however exhibit a lower basal GTPase activity than Drp1 WT in solution suggesting that the orientation of the VD, which does not directly participate in self-assembly, relative to the stalk imposes the helical geometry of Drp1 self-assembly and directs inter-rung G-domain dimerization for cooperative GTPase activity. Thus, we propose that the Drp1 VD akin to the dynamin PH domain^28^ acts a ‘fulcrum’ or a ‘constraint’ around which Drp1 stalk-mediated self-assembly and G-domain dimerization occur in the proper helical register.

We show that stable Drp1-Mff interactions, which likely do not involve the VD^36^, and target phospholipid (CL) binding, which specifically involves the VD, as demonstrated here and elsewhere^30^, alleviates VD-imposed auto-inhibition by effectively preventing the VD from dynamically accessing an intramolecular interaction site or interface, which when VD-bound in solution presents a steric blockade to helical self-assembly (Fig. 8). Mff- and/or CL-bound VD now constrained in dynamics promotes Drp1 helical self-assembly and stabilizes inter-rung G-domain dimerization for enhanced cooperative GTPase activity. Using the isolated VD as a tool, we demonstrate the existence of such an intramolecular VD interaction site vacated by the intrinsic VD upon Drp1-Mff or -CL interactions. We establish that the reoccupation of this interaction site by the isolated VD, mimicking the auto-inhibitory conformation of the intrinsic VD, selectively perturbs the longitudinal, inter-rung interactions of the Drp1 helical polymer, including G-domain dimerization, resulting in the suppression of cooperative GTPase activity. Thus, the VD specifically and allosterically regulates the inter-rung interactions of the distant GTPase domain along the long axis of the Drp1 helical polymer without significantly affecting the radial self-assembly of the connected Drp1 stalk.

Unlike prototypical dynamin that forms polymers of a uniformly narrow helical geometry on liposomes regardless of starting membrane curvature^73^, Drp1 forms curvature-adaptable helical polymers that more or less conform to the initial diameter of the target membrane template^35^. On membranes, Drp1 forms helical polymers of variable geometry that can range between ~30–200 nm in diameter^15,31^. We attribute this curvature adaptability to the VD. As opposed to the compact and globular dynamin PH domain^65^, the Drp1 VD remains largely unstructured in solution and samples an ensemble of conformations ranging from the compact to the highly distended as indicated by our results here. Moreover, the minimal ~111 aa VD region of Drp1 undergoes alternative splicing to include or exclude, either a part or whole of, an additional 37 aa region called the ‘B-insert’, to give rise to multiple Drp1 isoforms (splice variants). As Drp1 B-insert splice variants significantly differ in their diameter of helical self-assembly^15^, we postulate that the Drp1 VD, owing to its inherent flexibility, variable size, and a dynamic hydrodynamic volume (compressibility), directs Drp1 self-assembly in distinct geometries under varying conditions, such as in the presence of adaptors, target phospholipids (CL), varying membrane curvature etc. The VD is also the site of multiple post-translational modifications (e.g., sumoylation), which may further regulate Drp1 helical geometry and cooperative GTPase activity^74^.

Three of the four K residues in the Drp1 VD implicated in specific CL interactions are not conserved in yeast Dnm1p^75^. Yet, CL stimulates Dnm1p akin to mammalian Drp1^76^. We here demonstrate that these K residues are not directly involved in Drp1-CL interactions. Instead, they perturb the VD-mediated auto-inhibition of Drp1 higher-order self-assembly in solution. We show that a relative lack of membrane-active Drp1 dimers in solution in the case of Drp1 4KA, which favors higher-order self-assembly in solution, and a pronounced defect in the nucleotide-dependent assembly-disassembly dynamics for Drp1 R247A in solution, ostensibly diminishes the nucleation of Drp1 helical self-assembly by assembly-competent Drp1 dimers on membranes. The 4KA and the R247A mutants indirectly perturb Drp1-membrane interactions and self-assembly, and thus, fission.

The basal and assembly-stimulated GTPase activities of dynamin both play critical yet distinctive roles in endocytic vesicle scission^77^. The molecular mechanisms underlying the basal GTPase activity, and its functional consequences, however, have remained largely uncharacterized. We show here that the basal GTPase activity of the analogous Drp1 functions critically in the generation of membrane-active Drp1 dimers from higher-order oligomers in solution. Our data suggest that the Drp1 basal GTPase activity plays a critical role in shifting the solution oligomerization equilibria of Drp1 favorably toward assembly-competent dimers that can bind and nucleate higher-order helical self-assembly on target membranes (Fig. 8). We purport that a similar scenario likely occurs also in the case of dynamin. Although full-length dynamin exists as stable tetramers in solution both *in vitro* and *in vivo*^52,78^, it is mostly dynamin dimers, the minimal subunits of self-assembly, that nucleate higher-order helical self-assembly around endocytic pit necks for vesicle scission^79^. Based on our findings with Drp1, we speculate that the basal GTPase activity of dynamin functions similarly in the generation of membrane-active dynamin dimers from tetramers in solution. Thus, the dynamin and Drp1 basal GTPase activities function, respectively, as critical components of the endocytic and mitochondrial membrane fission pathways.

## Methods

### Protein purification and fluorescence labeling

DNA encoding C-terminal 6X His-tagged Drp1 short GG subcloned in pET21b (Novagen®)^17,80^ was a gift from Peter Göettig, University of Salzburg, Austria. The Drp1 long GG construct encoding the 13 additional amino acid residues of the 80-loop was created in the same vector by replacing a unique AatII-HindIII restriction fragment from the Drp1 short GG ORF with the corresponding fragment from Drp1 long^15^. The Δ80-loop deletion mutant in both full-length Drp1 short (short isoform; human isoform 3; 699 a.a.^15^) and Drp1 short GG was created by deleting residues 71–77 and 80–88 by mutagenic PCR. The two consecutive Drp1 T residues at positions 78 and 79, conserved between Drp1 and dynamin, were preserved for the β-turn. N-terminal 6X His-tagged Dyn1 GG and Dyn1 GG-PRD, linking residues 1–320 to 726–750 (GG) and residues 1–320 to 726–864 (GG-PRD), with an identical (GS)_4_ linker connecting the GTPase domain to the GED (BSE) region as for the Drp1 GG variants, were created anew using a similar strategy as reported in ref.^80^, and subcloned in pET28a (Novagen®). Full-length Dyn1 and Dyn1ΔPRD were also subcloned in pET28a. All full-length and truncation/deletion variants of both Drp1 and Dyn1 were expressed in *E*. *coli* BL21 star™ (DE3) as described earlier^80^ and purified to homogeneity using a protocol identical to that described for full-length human Drp1 and Drp1ΔVD (Δ514–602) previously^35,48^. Full-length Drp1-long-80-loop reported here corresponds to the Drp1-A-only construct described previously^15^. N-terminal 6X His-tagged Drp1 VD (residues 497–607) subcloned in pET28a (Novagen®) was expressed and purified likewise^35^. GST-tagged Drp1 VD (residues 497–607) and MffΔTM (residues 1–217) subcloned in pGEX6P-1 (GE Healthcare Lifesciences, Pittsburgh, PA) was prepared according to standard procedures, and the GST moiety was removed by excision with Pierce™ HRV 3 C Protease (ThermoFisher Scientific, Rockford, IL) according to the manufacturer’s protocols. The Dyn1 PH domain and MffΔTMΔCC were prepared as described earlier^28,36^. All DNA constructs were confirmed by automated DNA sequencing. It is important to note that the dimeric MffΔTM variant (1–217 aa) used in this study is different from the tetrameric MffΔTM variant (1–218 a.a.) described earlier^36^.

Full-length Mff was prepared also as previously described^15^, with multiple modifications to the purification protocol based on established procedures^81,82^ to obtain a greater purity and yield as elaborated in detail below. GST-tagged full-length Mff was expressed in *E*. *coli* BL21 Star™ (DE3) (ThermoFisher Scientific). Cells from an overnight pilot culture (50 ml) were transferred to fresh media (1 L) and grown to an OD600 of ~0.8–1.0 at 37 °C. The culture was then cooled on ice for 30 min, induced with 0.5 mM IPTG, and incubated with shaking for additional ~16 hours at 16 °C before harvesting. Cell pellets were resuspended in 20 ml of ice-cold Buffer A (20 mM HEPES, pH 7.5, 150 mM KCl) that contained 1% (v/v) Triton-X-100, 1 mM DTT, 1 mM AEBSF and a solubilized EDTA-free protease inhibitor cocktail tablet (Roche, Basel, Switzerland). Lysozyme was added to a final concentration of 1 mg/ml to the cell suspension and rocked gently for 30 min at 4 °C. Complete lysis was achieved by sonication. The lysate was clarified by centrifugation at 14,000 rpm in a Beckman Coulter JA-20 rotor for 45 min at 4 °C.

A 1 ml bed volume of Glutathione Sepharose 4B resin (GE Healthcare Lifesciences, Pittsburgh, PA) rinsed in Buffer A containing 0.1% (v/v) Triton-X-100 and 1 mM DTT was added to the clarified lysate and rocked for 1.5 hrs at 4 °C. Protein-bound resin was batch-washed thoroughly (20 ml × 3) with Buffer A containing 0.1% (v/v) Triton-X-100 and 1 mM DTT via low-speed centrifugation at 500×g in a Beckman Coulter JS 5.3 rotor. Protein-bound resin was then settled by gravity flow through a fritted column and washed with an additional 10 ml of the same buffer. GST-tagged full-length Mff was subsequently eluted with 5 ml of Buffer B containing 50 mM Tris, pH 8.0, 150 mM KCl, 1 mM DTT, 15 mM reduced glutathione (GSH) and 34 mM (1%) n-octyl-β-D-glucopyranoside (OG; SOL-GRADE®; Anatrace, Maumee, OH).

The N-terminal GST tag was excised from Mff by digesting the eluate with Pierce™ HRV 3 C Protease overnight on ice in the presence of 1 mM DTT and 1 mM EDTA, pH 8.0. Free GSH was removed by gel filtration of the eluate through disposable PD-10 desalting columns (2.5 ml × 2) equilibrated with Buffer A containing 1% OG. The excised GST was removed using a 0.5 ml bed volume of Glutathione Sepharose 4B resin also rinsed in Buffer A containing 1% OG, as above.

The flow-through containing full-length Mff was then purified to apparent homogeneity by passing the eluate through a 1 ml bed volume of charged Q Sepharose anion-exchange resin (GE Healthcare Lifesciences) pre-equilibrated with Buffer A containing 1% OG to trap remnant protein contaminants. Full-length Mff was estimated to be >90% pure by SDS-PAGE and Coomassie staining, and was stored at −80 °C in Buffer A containing 1% OG and 10% glycerol.

Full-length Drp1 and Drp1 VD were labeled with a thiol-reactive derivative of BODIPY-FL (ThermoFisher Scientific) as described previously^35^.

### Preparation of lipid templates

Liposomes were prepared by extrusion through 400-nm diameter polycarbonate membranes as described previously^35^. NT and GUVs were prepared by sonication and electroformation, respectively, as described earlier^28,35,48^. Unless otherwise specified, CL-containing liposomes and GUVs were composed of 25 mol% bovine heart CL, 35 mol% DOPE and ~40 mol% DOPC. CL-free membrane templates used for Drp1 (NT or liposomes) contained either 65 mol% DOPC (in liposomes), or 25 mol% DOPC and 40 mol% C24:1 β-D-galactosylceramide (Galcer; in NT), in addition to 35 mol% DOPE. Rhodamine-PE (RhPE), replacing an equivalent mole fraction of DOPC, was present at 0.1 mol% for GUV visualization. For Trp-Dansyl FRET measurements, dansyl-PE was present at 10 mol% in CL-containing liposomes that also contained 35 mol% DOPE and 30 mol% DOPC. For Drp1 VD (BODIPY-Fl)-liposome (RhPE) FRET experiments, DOPE was replaced by DOPC, and RhPE was present at 1 mol%. Liposomes used in ITC measurements likewise did not contain DOPE. PIP_2_-containing NT were composed of 10 mol% PIP_2_, 40 mol% Galcer and 50 mol% DOPC.

### Mff membrane reconstitution

Purified Mff was reconstituted in CL-free NT or liposomes at a protein/lipid molar ratio of 1:100 based on established procedures^81,82^. This was accomplished by mixing purified Mff stored in Buffer A containing 1% OG with NT or liposomes prepared in Buffer A. The Mff-NT/-liposome mixture was incubated for 10 min at room temperature and diluted 2-fold in Buffer A to lower the OG content below its critical micelle concentration. OG was removed by dialysis overnight at 4 °C against Buffer A.

### GTPase assay

GTP hydrolysis was measured using a malachite green-based colorimetric assay as previously described^35,83^. Released inorganic phosphate (P_i_) was monitored to obtain GTP hydrolysis rates and the turnover constant, *k*_cat_. The final concentration of GTP was 1 mM. Unless noted otherwise, the basal GTPase activity of full-length Drp1 was measured at 0.5 μM protein final. For CL- and Mff-stimulated GTPase activities, full-length Drp1 or mutants (0.5 μM final) were preincubated with the lipid templates (150 and 100 μM total lipid final, respectively, for CL-containing- and CL-free, Mff-containing lipid templates) prior to initiation of GTP hydrolysis. In experiments involving Mff-NT or -liposomes, the molar ratio of Drp1/Mff was 1:2 (1:200::Drp1:lipid). Dyn1 GTP hydrolysis in the absence and presence of PIP_2_-containing NT was measured at 0.5 μM protein final upon preincubation with 150 μM final lipid. GTP hydrolysis under low ionic-strength conditions were performed in buffer containing 20 mM HEPES, pH 7.5, 1 mM DTT and <25 mM KCl. Data points typically represent averages of three independent experiments ± S.D.

### SEC-MALS

SEC elution profiles, differential refractive indices, and molar mass distributions were obtained as described previously^35^. Unless otherwise noted, full-length Drp1 WT and mutants were each loaded at 10 μM in a 500 μl volume onto a Superose 6 10/300 GL column (GE Healthcare Lifesciences) pre-equilibrated with Buffer A containing 1 mM DTT. Drp1 short GG and Drp1 long GG at 25 μM each, and Dyn1 GG and Dyn1 GG-PRD at 15 μM each, after 30 min room temperature incubation in the absence and presence of the transition-state analog (GDP.AlF_x_), generated from a mixture of 2 mM GDP, 4 mM MgCl_2_, 2 mM AlCl_3_, and 20 mM NaF, in a final volume of 500 μl, were fractionated through a Superdex 75 10/300 GL column (GE Healthcare Lifesciences) also maintained at room temperature. Δ80-loop Drp1 GG was fractionated at a loading concentration of 6 μM in comparison to Drp1 short GG at an equivalent concentration, similarly. GST-excised, tag-free Drp1 VD (45, 75 or 200 μM), Dyn1 PH domain (40 μM), Mff ΔTM (100 μM), Mff ΔTMΔCC (75 μM)^36^, and GST (100 μM) were fractionated through either a Superdex 75 or a Superose 6 10/300 GL column as noted in the respective figure panels. Representative elution traces and molar mass profiles are shown.

### Electron microscopy

Negative-stain EM of Drp1 (2 μM final) either upon incubation with GMP-PCP (1 mM final) in solution, or upon incubation with 25 mol% CL-containing liposomes or NT (50 μM total lipid final), in the absence and presence of the isolated Drp1 VD (170 μM final) or GTP (1 mM final) was performed as described previously^35,48^. Drp1ΔVD polymers in suspension or upon sedimentation were imaged similarly. Dyn1 (2 μM final) incubated with 10 mol% PIP_2_-containing NT (50 μM total lipid final), in the absence and presence of the isolated Dyn1 PH domain (150 μM final), was also imaged likewise.

### Spin sedimentation assay

Drp1 WT (2 µM protein final) was incubated with 25 mol% CL-containing NT (200 µM lipid final) in Buffer A in the absence and presence of a vast molar excess of Drp1 VD (50 μM protein final) for 30 min at room temperature. Supernatant (S) and pellet (P) Drp1 fractions of these samples were obtained by high-speed centrifugation of the samples at 20,800 × g in a refrigerated micro-centrifuge maintained at 4 °C, and visualized by Coomassie staining following SDS-PAGE.

### Confocal imaging of GUVs

All measurements were made at 25 °C using methods and instrumentation as previously described^35,48^. Representative images are shown.

### Secondary structure prediction

The secondary structure of Drp1 VD (residues 497–607) was predicted using the PSIPRED v3.3. software^84^ available online at http://bioinf.cs.ucl.ac.uk/psipred/.

### Circular dichroism spectroscopy

Far-UV CD spectra were recorded between 190–280 nm in 0.5 nm increments using an Aviv Model 215 CD spectropolarimeter temperature-equilibrated at 25 °C. 6 X His-tagged Drp1 VD was present at 200 μM final, whereas MffΔTM was present at 17.2 μM final, both in PBS, pH 7.4. Representative spectra are shown.

### FRET

Trp (donor)-dansyl (acceptor) FRET was monitored upon the rapid mixing of 0.4 μM Drp1 with 25 mol% CL-containing liposomes (20 μM total lipid) also containing 10 mol% dansyl-PE, at 25 °C in a Fluorolog 3–22 spectrofluorometer (HORIBA) outfitted with a SFA-20 Rapid Kinetics Stopped-Flow Accessory (Hi-Tech Scientific, Bradford-on-Avon, UK). Drp1 Trp was excited at 280 nm and dansyl emission was monitored at 530 nm using excitation and emission slit-widths of 2 and 12 nm, respectively. FRET between BODIPY-FL (donor)-labeled Drp1 VD and rhodamine-PE (acceptor)-labeled liposomes was measured using a Tecan Infinite M1000 PRO microplate reader maintained at room temperature. BODIPY-FL was excited at 470 nm and the FRET-sensitized rhodamine emission intensity was monitored at 590 nm. The final concentration of total lipid in the experiments was 2.5 mM. FRET efficiency (*E*) was calculated using the equation, *E* = 1 − (*F*_DA_/*F*_D_), where *F*_DA_ is the emission intensity of BODIPY-FL monitored at 510 nm in the presence of rhodamine-PE, and *F*_D_ is the corresponding emission intensity in the absence of rhodamine-PE in the liposomes. For the measurement of E as a function of fractional CL content, BODIPY-FL-labeled Drp1 VD was used at a final concentration of 27.5 μM.

### Isothermal titration calorimetry

Binding of 6X His-tagged Drp1 VD to 50 mol% CL-containing liposomes, or to control 100% DOPC liposomes, was measured at 25 °C in Buffer A using a MicroCal ITC200 calorimeter (GE Healthcare Lifesciences). Liposomes at a total lipid concentration of 5 mM were titrated through successive injections into the sample cell containing Drp1 VD (2 μM final) under constant stirring. The binding isotherms were analyzed using the Origin® statistical software package to obtain the equilibrium-binding constant, *K*_*D*_. Representative traces are shown.

### Dynamic light scattering (DLS)

DLS measurements of protein hydrodynamic radius (diameter) and polydispersity were performed in a Dynapro Nanostar (Wyatt Technologies) instrument similar to previously published methods^85^. 50 μl samples of either untagged Drp1 VD or Dyn1 PH at 90 μM final each were analyzed in an Eppendorf UVette at room temperature. Autocorrelation curves from a set of ten acquisitions (10 sec integration time each) were analyzed using the Dynamics v7.1.3 software (Wyatt Technologies) to resolve the oligomerization or conformational states within each sample. The Dyn1 PH monomer intensity autocorrelation trace was best fit (red trace) to a single species that corresponded well to the hydrodynamic radius of the crystallized Dyn1 PH domain monomer^65^, whereas the Drp1 VD monomer trace was unable to be best fit to a single species, and was interpreted to correspond to an ensemble of various conformational states.

### 90° light scattering

Drp1 WT and mutants at 1 μM each in a total final volume of 400 μl were equilibrated in Buffer A containing 2 mM MgCl_2_ and 1 mM DTT in a 4 mm × 4 mm quartz cuvette (Starna cells, CA) temperature-equilibrated at 25 °C. Scattered light from these samples was measured continuously at 350 nm (0.5 nm bandpass; 0.5 sec integration time) using a Fluorolog 3–22 photon-counting spectrofluorometer (Horiba, NJ), before and after addition of GMP-PCP or GTP to a final concentration of 1 mM (marked by arrows) at a defined time point. The intensity traces were corrected for dilution upon addition of nucleotide.

### Cell biology

Mitochondrial morphology and distribution in Drp1-*null* mouse embryonic fibroblasts (Drp1-KO MEFs) expressing either Drp1 WT or Δ80-loop Drp1 were analyzed as previously described^35,48^. Drp1 co-localization on mitochondria was quantified as described elsewhere^36^. Drp1 expression levels were detected and compared using western blotting also as previously described^35^.

## Electronic supplementary material

Supplementary Information
