# Supplementary material for: Steric interference from intrinsically disordered regions controls dynamin-related protein 1 self-assembly during mitochondrial fission
Source: Sci Rep. 2018 Jul 18;8:10879. doi: 10.1038/s41598-018-29001-9 (PMC6051998; doi:10.1038/s41598-018-29001-9)

# Steric interference from intrinsically disordered regions controls dynamin-related protein 1 self-assembly during mitochondrial fission

**Bin Lu[1, *], Bridget Kennedy[1, *], Ryan W. Clinton[2,3,4], Emily Jue Wang[1], Daniel McHugh[1], Natalia Stepanyants[1], Patrick J. Macdonald[1], Jason A. Mears[2,3,4], Xin Qi[1,3], and Rajesh Ramachandran[1,3,4, ¶]**

**Supplementary Information**

**Supplementary Figure S1. Comparable behavior of full-length Drp1 and Dyn1 variants to the corresponding GG constructs. A)** Basal GTPase activity of Drp1-short 80-loop and Drp1-long 80-loop measured as a function of protein concentration. **B)** Assembly-stimulated GTPase activity of Drp1-short 80-loop and Drp1-long 80-loop under low ionic-strength conditions (<25 mM KCl) measured as a function of protein concentration. **C)** CL-stimulated GTPase activity of Drp1-short 80-loop and Drp1-long 80-loop upon incubation with 25 mol% CL-containing liposomes (150 μM lipid final) measured as a function of protein concentration. % of maximum GTPase activity for each variant is plotted. *Inset* shows $k_{cat}$ plotted for the same. **D)** Basal GTPase activity of Dyn1 and Dyn1ΔPRD measured as a function of protein concentration. **E)** Assembly-stimulated GTPase activity of Dyn1 and Dyn1ΔPRD under low ionic-strength conditions (<25 mM KCl) measured as a function of protein concentration.

**Supplementary Figure S2. Myc-tagged Drp1 WT and Δ80-loop Drp1 were expressed to equivalent levels in Drp1 KO MEFs.** Uncropped western blots for images shown in Fig. 1L (boxed). Lanes are denoted with their respective sample contents. Actin served as loading control. Set 2 (duplicate) was loaded in the same order as set 1 (boxed).

**Supplementary Figure S3. Δ80-loop Drp1 is impaired in self-assembly and cooperative GTPase activity. A and B)** Representative EM images of full-length Δ80-loop Drp1 upon incubation with either GMP-PCP (panel **A**) or CL-NT (panel **B**), in comparison to Drp1 WT. Scale bar, 200 nm. *Inset* scale bar, 50 nm. **C)** Basal and CL-stimulated GTPase activities of full-length Δ80-loop Drp1 in comparison to Drp1 WT.

**Supplementary Figure S4. Isolated Drp1 VD does not impair full-length Drp1 WT-membrane interactions.** Uncropped Coomassie brilliant blue-stained SDS-PAGE gel from which cropped images shown in Fig. 5G (boxed) were isolated. Lanes are denoted with their respective sample contents. Note that the isolated Drp1 VD was not pelleted considerably along with Drp1-bound CL-NT due to the apparently weak and reversible interaction with full-length Drp1 WT.

# Supplementary Figure S1

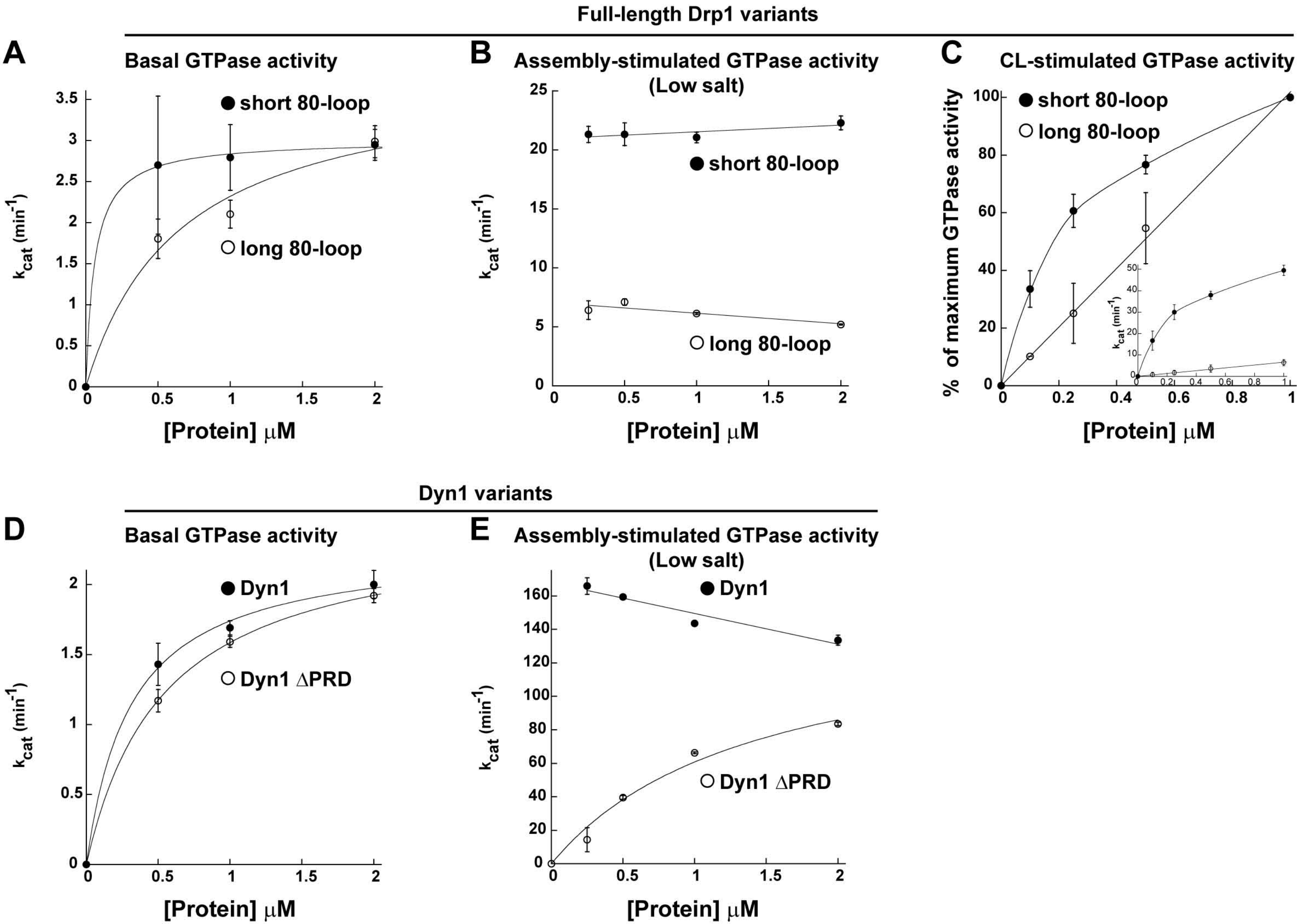

# Supplementary Figure S2

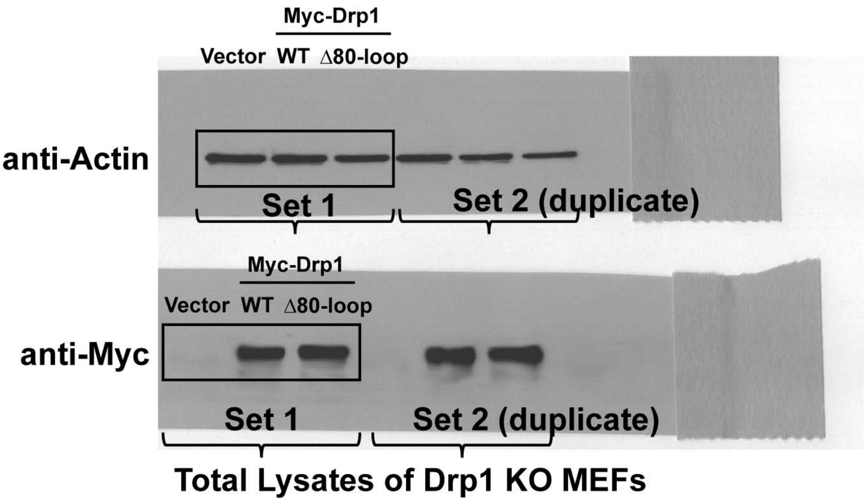

# Supplementary Figure S3

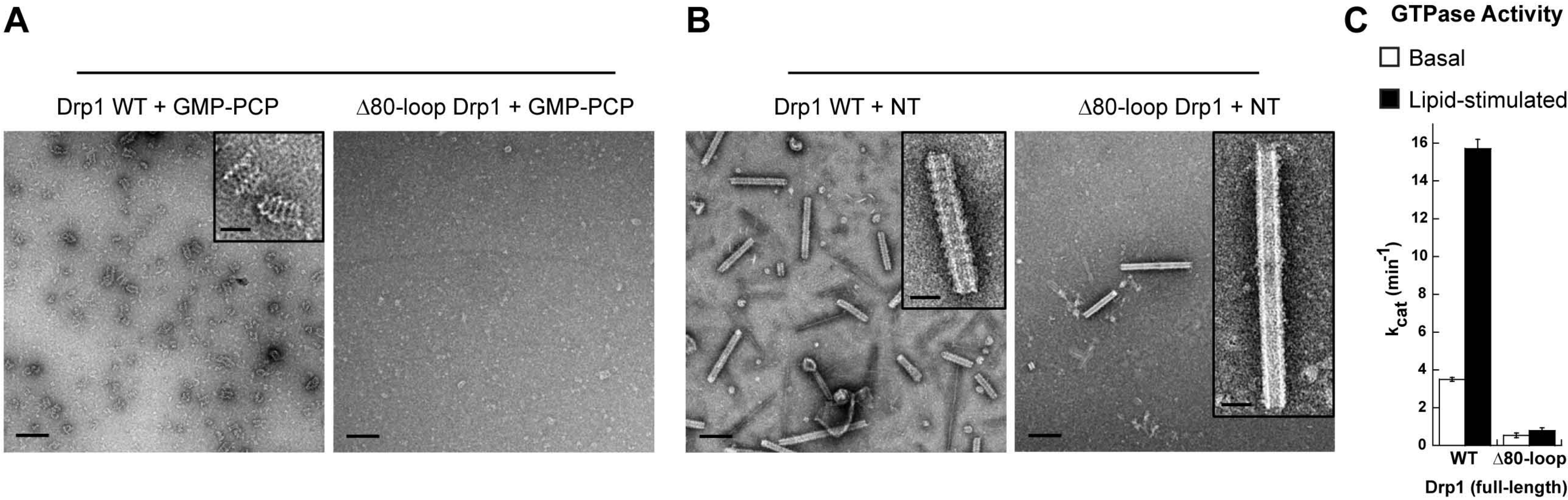

# Supplementary Figure S4

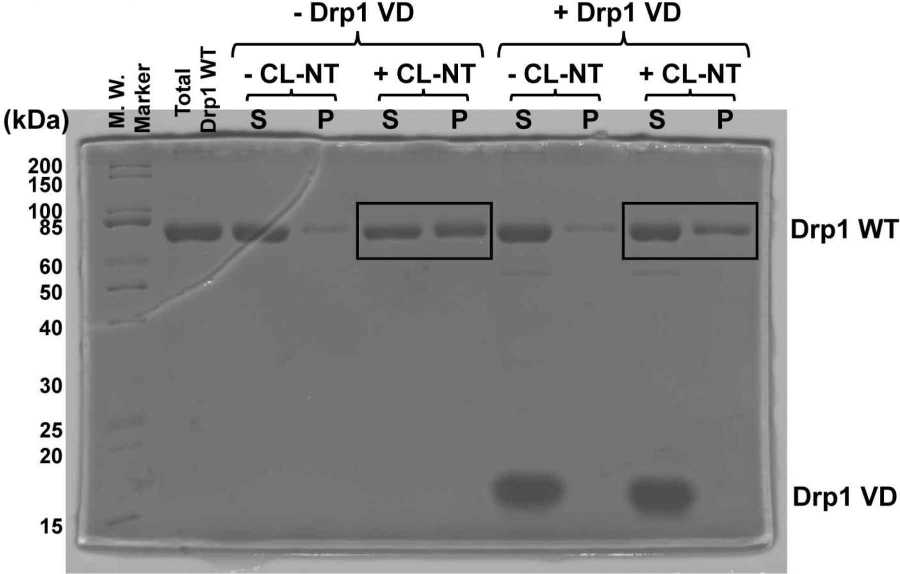

Supplement: Supplementary file 1 — Supplementary Information [file 41598_2018_29001_MOESM1_ESM.pdf]
